# The DNA Damage Response Pathway Contributes to the Stability of Chromosome III Derivatives Lacking Efficient Replicators

**DOI:** 10.1371/journal.pgen.1001227

**Published:** 2010-12-02

**Authors:** James F. Theis, Carmela Irene, Ann Dershowitz, Renee L. Brost, Michael L. Tobin, Fabiana M. di Sanzo, Jian-Ying Wang, Charles Boone, Carol S. Newlon

**Affiliations:** 1 Department of Microbiology and Molecular Genetics, New Jersey Medical School, University of Medicine and Dentistry of New Jersey, Newark, New Jersey, United States of America; 2 Banting & Best Department of Medical Research and Department of Molecular Genetics, Terrence Donnelly Centre for Cellular and Biomolecular Research, University of Toronto, Toronto, Ontario, Canada; National Cancer Institute, United States of America

## Abstract

In eukaryotic chromosomes, DNA replication initiates at multiple origins. Large inter-origin gaps arise when several adjacent origins fail to fire. Little is known about how cells cope with this situation. We created a derivative of *Saccharomyces cerevisiae* chromosome III lacking all efficient origins, the 5ORIΔ-ΔR fragment, as a model for chromosomes with large inter-origin gaps. We used this construct in a modified synthetic genetic array screen to identify genes whose products facilitate replication of long inter-origin gaps. Genes identified are enriched in components of the DNA damage and replication stress signaling pathways. Mrc1p is activated by replication stress and mediates transduction of the replication stress signal to downstream proteins; however, the response-defective *mrc1^AQ^* allele did not affect 5ORIΔ-ΔR fragment maintenance, indicating that this pathway does not contribute to its stability. Deletions of genes encoding the DNA-damage-specific mediator, Rad9p, and several components shared between the two signaling pathways preferentially destabilized the 5ORIΔ-ΔR fragment, implicating the DNA damage response pathway in its maintenance. We found unexpected differences between contributions of components of the DNA damage response pathway to maintenance of ORIΔ chromosome derivatives and their contributions to DNA repair. Of the effector kinases encoded by *RAD53* and *CHK1*, Chk1p appears to be more important in wild-type cells for reducing chromosomal instability caused by origin depletion, while Rad53p becomes important in the absence of Chk1p. In contrast, *RAD53* plays a more important role than *CHK1* in cell survival and replication fork stability following treatment with DNA damaging agents and hydroxyurea. Maintenance of ORIΔ chromosomes does not depend on homologous recombination. These observations suggest that a DNA-damage-independent mechanism enhances ORIΔ chromosome stability. Thus, components of the DNA damage response pathway contribute to genome stability, not simply by detecting and responding to DNA template damage, but also by facilitating replication of large inter-origin gaps.

## Author Summary

Loss of genome integrity underlies aspects of aging and human disease. During DNA replication, two parallel signaling pathways play important roles in the maintenance of genome integrity. One pathway detects DNA damage, while the other senses replication stress. Both pathways activate responses that include arrest of cell cycle progression, giving cells time to cope with the problem. These pathways have been defined by treating cells with compounds that induce either replication stress or DNA damage, but little is known about their roles during unperturbed DNA replication. They may be important when several adjacent replication origins fail to initiate and forks from flanking origins must replicate longer regions of DNA than normal to complete replication. We have used a derivative of budding yeast chromosome III lacking all efficient replication origins to identify mutants that preferentially destabilize this chromosome fragment, which mimics a chromosome with a large inter-origin gap. We found that the DNA damage response pathway, but not the replication stress response pathway, plays an important role in maintaining this fragment. The signal recognized in this case may be replisome failure rather than forks stalled at endogenous DNA damage.

## Introduction

In eukaryotic chromosomes, DNA replication initiates at multiple origins, specified by *cis*-acting sequences called replicators. In the budding yeast, *Saccharomyces cerevisiae*, replicators are termed ARS elements and were identified by their ability to promote extrachromosomal maintenance of plasmids. Chromosomal replication origins coincide with ARS elements, which contain the binding site for the six-subunit initiator complex, ORC. During G1, ORC recruits additional proteins to form pre-replicative complexes (pre-RCs) that initiate replication during S phase [1]. The average distance between active replication origins in *S. cerevisiae* is approximately 40 kb, based on both electron microscopic analysis of replicating DNA molecules [2] and whole genome analysis [3], [4]. In fission yeast, a similar range of estimates was obtained from whole genome analysis and DNA combing [3], [5].

The presence of multiple origins on chromosomes raises the question of whether replicators are activated according to a fixed temporal program or whether their use is stochastic, i.e. different replicators are activated in different cells or in successive S phases. In budding yeast, 2D-gel analyses and replication timing studies suggested that replicators are activated according to a program, although some variability is inevitable because some replicators fire inefficiently [3], [4], [6]–[12]. Recent single-molecule studies in budding yeast ([13], [14] Wang and Newlon, manuscript in preparation), and in fission yeast [5], [15]–[18] reflect this stochasticity in initiation.

Stochastic activation of replicators should occasionally produce large inter-origin gaps caused by failure of adjacent origins to initiate, referred to as the random gap problem [19]. Recent theoretical analysis of the replication dynamics of the fission yeast genome based on data that describe the positions and firing probabilities of replicators and the rate of fork movement suggests that long inter-origin gaps occur frequently in fission yeast [20]. In 88% of 2000 simulations using this stochastic hybrid model, at least one region of the genome contained an inter-origin gap more than 6-fold longer than the average inter-origin spacing; replication of such a gap would require about twice the known length of S phase. These results suggest that completion of DNA replication requires most of the normal G2 period of the cell cycle, and in some fraction of the population, regions of the genome would still be replicating at the normal time of mitosis. The problematic regions included about 5% of the genome, and each individual region appeared infrequently in the simulations, making such regions difficult to detect experimentally. It is not known how cells cope with this issue.

**Figure 1:**
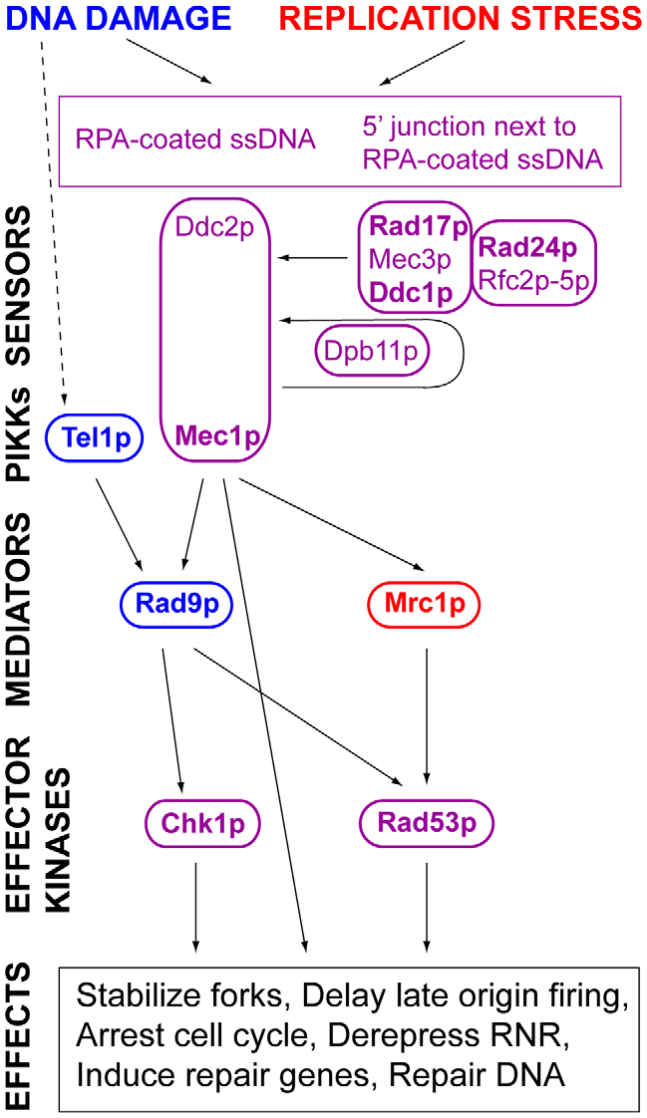
DNA damage and replication stress response pathways. A simplified version of the DNA damage and replication stress checkpoint pathways is shown. The pathways are conceptually divided into sensors, phosphoinosotide-3-kinase-related kinases (PIKKs), mediators and effector kinases. The shared components of the pathways are shown in purple. The pathway-specific mediators, Rad9p, and Mrc1p, are shown in blue and red, respectively. The pathways are activated by sensors. Mec1p and Ddc2p form a complex, homologous to the mammalian ATR-ATRIP complex, which recognizes Replication Protein A (RPA) bound to ssDNA [88]. Rad17p, Mec3p, and Ddc1p form a PCNA-like complex, homologous to the 9-1-1 complex, which is loaded onto DNA at 5′ junctions adjacent to single-stranded DNA coated with RPA by an alternative clamp loader in which Rad24p replaces Rfc1p in a complex with Rfc2p, Rfc3p, Rfc4p, and Rfc5p [89]–[92]. Binding of the Rad17p-Ddc1p-Mec3p clamp results in activation of Mec1p kinase activity. Ddc1p is phosphorylated by Mec1p [90]. Dpb11 binds to phosphorylated Ddc1p and mediates a more robust activation of Mec1p [93]. Signals from the PIKK kinases are transduced to effector kinases with the help of mediators (see text). Components tested are shown in bold type.

One possibility is that ongoing replication activates a checkpoint response to prevent cells from undergoing mitosis prior to completion of S phase. Two intertwined checkpoints function during S phase (Figure 1). The DNA damage response is activated by a signal transduction cascade in response to stalling of replication forks encountering DNA damage (reviewed by Branzei and Foiani [21], [22]). Experimentally, this response is activated by treatment with MMS or UV; unperturbed cells probably activate this pathway in response to forks encountering endogenous DNA damage. The replication stress response is activated experimentally by hydroxyurea treatment, which slows replication forks by inhibiting ribonucleotide reductase; it is not known what endogenous signal(s) activate(s) it.

Activation of an S phase response may occur in cells coping with long inter-origin gaps. Rad53p, the ortholog of the mammalian and fission yeast effector kinase, Chk2 (Figure 1), becomes hyperphosphorylated late in S phase in mutants that fail to fire some replication origins, indicating activation of a checkpoint [23], [24]. In addition the stability of a yeast artificial chromosome (YAC) carrying human DNA sequences from which origins had been deleted depended on *RAD9*, the mediator in the DNA damage response pathway [25]. However, other evidence suggests that cells do not monitor either the initiation or completion of DNA replication. For example, strains carrying tight alleles of *cdc6* (*cdc18^+^* in *S*. *pombe*), which encodes a pre-RC component, or of *dbf4*, the regulatory subunit of the Cdc7p kinase required for origin firing, proceed directly from G1 to mitosis despite failing to replicate any DNA [26]–[28]. Even ongoing replication may not prevent anaphase entry [29], [30].

**Figure 2:**
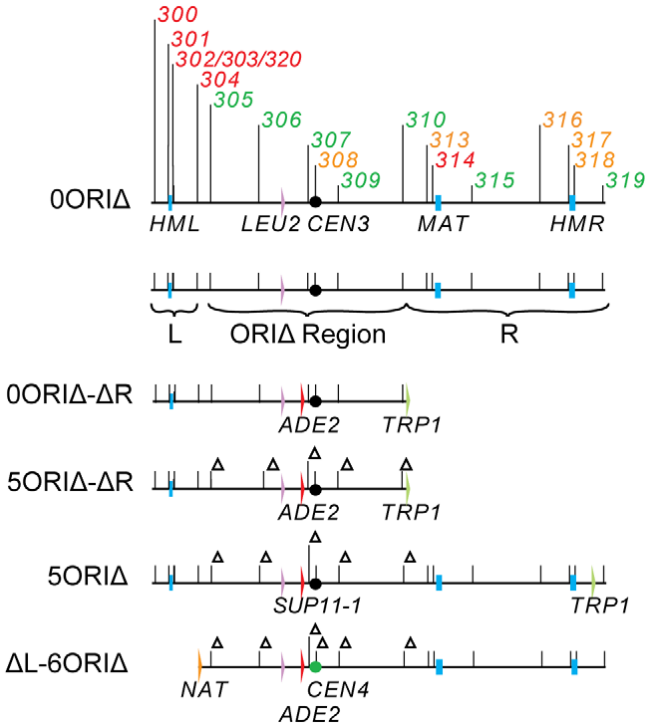
Chromosome III derivatives. The diagram at the top summarizes replicator activity on the wild type (0ORIΔ) chromosome. ARS elements are numbered above the line and color coded to indicate efficiencies: green, active in ≥90% of cell cycles; yellow, active in 15–25% of cell cycles; red, not detectably active [55]. The diagram below shows regions altered in ORIΔ derivatives; individual deletions were made in the ORIΔ region, and the number of deletions present is specified by a number, e.g. 0ORIΔ (no origins deleted) or 5ORIΔ (the efficient origins deleted). Additional ORIΔ derivatives were made by fragmenting the chromosome just to the right of *ARS304* to remove dormant origins in the ‘L’ region, or just to the right of *ARS310* to remove origins in the ‘R’ region. We refer to these derivatives as ΔL-ORIΔ and ORIΔ-ΔR derivatives. Blue boxes indicate the positions of the *HML*, *MAT* and *HMR* loci. The lavender arrows indicate the position of the *LEU2* gene; the red arrows indicate the position of the *ADE2* or *SUP11-1* insert; the filled black circles indicate *CEN3*; the green filled circle indicates the *CEN4* replacement of *CEN3*, which removes *ARS308*; green arrows indicate the positions of *TRP1* inserts; the orange arrow indicates the position of the *NAT1* insert.

We have created a derivative of yeast chromosome III lacking efficient replicators as a tool to detect mechanisms that contribute to replication of large inter-origin gaps (the 5ORIΔ-ΔR fragment - Figure 2). This fragment is composed entirely of yeast sequences with the exception of plasmid sequences at the fragmentation point. It replicates efficiently, with a loss rate per division of 2.1×10^−3^ [31] and is much more stable than the YAC [25]. We carried out a genetic screen for mutants specifically defective in maintenance of this ORIΔ derivative on the premise that mutations that caused destabilization of the 5ORIΔ-ΔR derivative, but had little or no effect on maintenance of the corresponding 0ORIΔ-ΔR derivative (Figure 2) would identify genes required for the replication of long inter-origin gaps, or perhaps new replication initiation mechanisms. This screen identified three originless fragment maintenance (Ofm) mutants, one dominant, *OFM1-1*, and two recessive, *ofm6-1* and *ofm14* (an allele of *RAD9*) [32]. The *rad9* mutation increased the loss rate of the 5ORIΔ-ΔR fragment, but did not cause the frequent rearrangement that was seen with the YAC [32].

Here we report the results of a modified synthetic genetic array (SGA) screen [33], [34] used to identify additional Ofm mutants. Deletions of several genes in the DNA damage response pathway caused an Ofm phenotype. Further analysis indicated that this pathway contributes to the replication of large inter-origin gaps. In contrast, the replication stress response pathway does not contribute to the stability of the 5ORIΔ-ΔR fragment. Surprisingly, genes in the homologous recombination pathway, which are believed to contribute to the restart of collapsed replication forks, do not contribute to the maintenance of the fragment.

## Results

### SGA+Chromoduction-based screen for Ofm mutants

Our previous visual screen for Ofm mutants was labor intensive, during both the initial visual screening of colonies grown from the mutagenized culture and in subsequent attempts to identify mutations responsible for the phenotype. Thus, we adapted synthetic genetic array (SGA) technology [33], [34] for use in a colony sectoring screen to identify additional Ofm mutants in the *S. cerevisiae* viable deletion collection. One limitation of this screen is that essential genes could not be tested.

**Figure 3:**
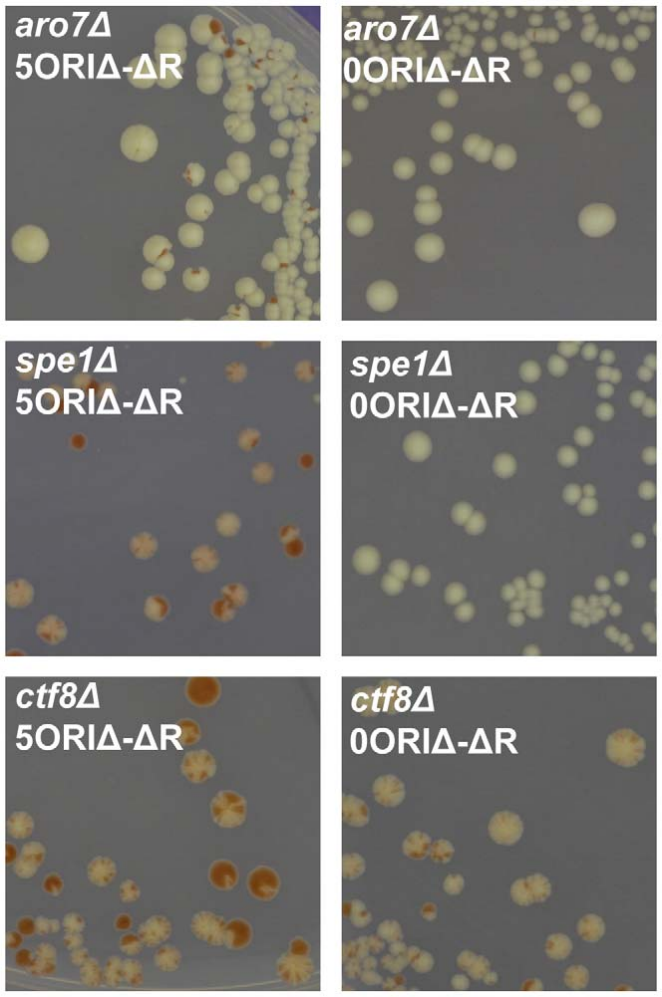
Examples of sectoring patterns. In the primary screen the 5ORIΔ-ΔR fragment (marked with *ADE2* and *LEU2*) was introduced into each of the *ade2Δ::natR xxxΔ::kanR* double mutants by chromoduction. Chromoductants were streaked on medium with limiting adenine. Chromosome loss events appear as red sectors due to the accumulation of a pigment in *ade2* mutants. For the secondary screen, sectoring colonies from the primary screen were re-streaked. The 5ORIΔ-ΔR and 0ORIΔ-ΔR fragments were separately introduced by chromoduction into a Leu^−^ Ade^−^ colony from these streaks. These chromoductants were then streaked on limiting adenine medium and photographed after 5 days. Left panels: Photographs of mutants carrying the 5ORIΔ-ΔR fragment: *aro7Δ* - wildtype level of sectoring; *spe1Δ* - highly elevated sectoring; *ctf8Δ* - highly elevated sectoring. Right panels: Photographs of mutants carrying the 0ORIΔ-ΔR fragment. *spe1Δ* was classified as an Ofm mutant because colonies carrying the 0ORIΔ-ΔR fragment were rarely sectored. *ctf8Δ* was classified as a non-Ofm mutant because colonies carrying the 0ORIΔ-ΔR fragment were highly sectored. See also Table S1.

In the primary screen, as detailed in Methods, we used SGA technology to create *ade2Δ::natR xxxΔ::kanR* haploid *MAT* ***a*** progeny. We then used chromoduction [35] to introduce the 5ORIΔ-ΔR fragment of chromosome III marked with *ADE2* into each strain (Figure 2). Chromoductants, each carrying the 5ORIΔ-ΔR fragment were then streaked on plates with limiting adenine. Loss of the *ADE2-*marked fragment during growth of a colony results in a red sector. If a mutant has a low 5ORIΔ-ΔR fragment loss rate, such sectors will be rare; conversely, a mutant with an elevated loss rate will yield highly sectored colonies, providing a semi-quantitative estimate of loss rates. Examples of sectoring patterns are shown in Figure 3. The majority of the 5171 strains screened showed a low rate of sectoring as illustrated by the *aro7Δ* mutant used as a control. Ninety strains had an elevated rate of sectoring, as shown by the *spe1Δ* and *ctf8*Δ strains.

The elevated sectoring observed for the 90 strains selected from the primary screen could reflect either defects in transmission of all chromosomes, e.g. a defect due to the loss of a component of the kinetochore, or defects specific to 5ORIΔ-ΔR fragment transmission. To distinguish between these possibilities, we identified a colony from each of the 90 strains that had lost the 5ORIΔ-ΔR fragment, then separately introduced by chromoduction the 0ORIΔ-ΔR and the 5ORIΔ-ΔR fragments (Figure 2), and compared the sectoring phenotypes of two independent chromoductants carrying each of these fragments by estimating the number of red sectors per colony seen in chromoductants. Our previous measurements of loss rates of these chromosome III derivatives by fluctuation analysis allowed us to make semi-quantitative estimates of loss rates based on sectoring patterns [31], [32]. The loss rate of the 5ORIΔ-ΔR derivative is ∼2×10^−3^ losses per division in wild type cells, and this loss rate results in 0–3 sectors per colony in the SGA strain background. Colonies of strains carrying the 0ORIΔ-ΔR derivative, which has a loss rate of about 2×10^−5^ losses per division, rarely have a red sector. Mutant strains with loss rates of the 5ORIΔ-ΔR fragment in the range of 10^−2^ losses per division form colonies with 5–10 sectors per colony, and strains with loss rates in the range of 10^−1^ losses per division form colonies with ≥10 sectors per colony. The results of this secondary screen are detailed in Table S1. For example, *spe1Δ* was classified as an Ofm mutant because cells carrying the 5ORIΔ-ΔR fragment gave rise to colonies with 5–10 sectors per colony, while those carrying the 0ORIΔ-ΔR fragment yielded colonies that were rarely sectored; *ctf8Δ* was called a non-Ofm mutant because cells carrying either fragment gave rise to colonies with >10 sectors per colony (Figure 3). Overall, the 71 deletion strains in which the high sectoring phenotype of the 5ORIΔ-ΔR fragment was reproduced in the secondary screen were divided into high confidence Ofm mutants (52 strains), possible/probable Ofm mutants (14 strains) and non-Ofm mutants (5 strains) (Table 1). In the high confidence Ofm mutants, the two chromoductants carrying the 5ORIΔ-ΔR derivative were estimated to have at least 5–10 sectors per colony, and the two chromoductants carrying the 0ORIΔ-ΔR derivative rarely gave rise to a colony with a sector. In the case of the probable/possible Ofm mutants, either the two 5ORIΔ-ΔR chromoductants or the two 0ORIΔ-ΔR chromoductants showed different sectoring patterns. In the non-Ofm mutants, the 0ORIΔ-ΔR chromoductants all showed a sectoring pattern consistent with at least a 100-fold increase in the loss rate of this derivative.

**Table 1:** Genes identified in screen.

Ofm mutants	*asi2, ast2, bfa1, bre5, chd1, ctf18, ecm7, gut2, gyp1, hch1, hst3, idh1, idh2, ioc2, irc14, isw1, lsm1, mad2, mad3, mcr1, mid1, mtc1, mth1,pol32, puf3, rad9, rad17, rad24, rpa34, rpl20b, rpl34b, sdp1, sgf73, sgs1, sip3, skt5, sop4, spe1, spt8, swi5, ubp3, vph2, whi4, ybr099c, ydr278c, yer046w-a, yfr016c, yhl005c* 1, *yor024w* 2, *ypk1*
Possible/Probable Ofm mutants	*acm1, bim1, bub1, bub2, bub3, chl1, csm3, dia2, rmi1, rtg1,rtg3, sok2, tof1, top3* 3
Non-Ofm mutants	*ctf4, ctf8, kar3, mad1, sic1,*

1 YHL005C is a dubious ORF that partially overlaps *MRP4*, which encodes a mitochondrial ribosomal protein. It also occupies the promoter region of *SHU1*, which functions in a *RAD51* and *RAD54*-dependent pathway for homologous recombinational repair.

2 YOR024W is a dubious ORF upstream of *HST3*; *hst3Δ* was also scored as an Ofm mutant. *yor024wΔ* leaves only 52-base-pairs upstream of the *HST3* ORF intact, suggesting that this deletion alters *HST3* expression.

3 *top3* mutants are slow-growing and rapidly accumulate *sgs1* mutations which suppress the slow-growth phenotype [94]; the chromoductants screened are likely *top3 sgs1* double mutants. *sgs1* was scored as an Ofm mutant (see Table S1).

A gene ontology (GO) analysis was performed on the 52 genes whose deletion caused Ofm phenotypes and on the 5 genes whose deletion caused non-Ofm phenotypes (http://db.yeastgenome.org/cgi-bin/GO/goTermFinder.pl). The three highest scoring clusters among the Ofm mutants (P = 8×10^−5^–4×10^−3^) share many genes and correspond to the annotations “cell cycle checkpoint”, “DNA damage response, signal transduction”, and “DNA damage checkpoint”. The cell cycle checkpoint cluster (*SGS1, BFA1*, *MAD2, MAD3, RAD9*, *RAD17*, and *RAD24)* included all of the genes present in the other two clusters. When the possible/probable Ofm mutants were included in the analysis the highest scoring cluster was still “cell cycle checkpoint” (p = 8×10^−7^). In addition to the 7 genes above, the cluster included *BIM1*, *BUB1*, *BUB2*, *BUB3*, *CSM3* and *TOF1*. *RAD9*, *RAD17*, and *RAD24* function in the DNA damage response pathway while *MAD2* and *MAD3* function in the spindle checkpoint, though some results have suggested an additional role in the DNA damage checkpoint [36]–[38]. *BFA1* and *BUB2* are required to prevent mitotic exit in both the DNA damage and spindle checkpoint pathways [39]. The highest scoring cluster (P = 3×10^−6^) among the non-Ofm mutants corresponded to the annotation “mitotic cell cycle”. This cluster included all five mutants identified as non-Ofm mutants.

### Mutations in the DNA damage response pathway, but not the replication stress response pathway, cause an Ofm phenotype

Results of the GO analysis and identification of a null allele of *RAD9* in our forward mutation screen [32] led us to examine the DNA damage response pathway in more detail. We moved the deletions of interest into the YKN10 strain background (Table S2) as described in Methods. Analysis of these strains allowed us to confirm that each deletion caused an Ofm phenotype and to quantitate the effects of the mutations in the strain background with which we had the most experience.

Our premise in undertaking this screen is that problems with the replication of the 5ORIΔ-ΔR derivative may be qualitatively different than the problems sustained by the 0ORIΔ-ΔR derivative by virtue of the presence of a long inter-origin gap. Therefore, we wanted to be able to make a quantitative comparison of loss rates that are very different. We reasoned that a comparison of the number of additional loss events sustained by the 5ORIΔ-ΔR and 0ORIΔ-ΔR derivatives in a given mutant would provide a measure of the strength of the Ofm phenotype. We define the “Ofm index” as the number of additional loss events per 10^5^ divisions for the 5ORIΔ-ΔR derivative divided by the number of additional losses for the 0ORIΔ-ΔR derivative (Table 2). Two examples illustrate our reasoning. Suppose that in a wild type cell the loss rate of the 0RIΔ-ΔR derivative is 1 and the loss rate of the 5ORIΔ-ΔR derivative is 100. In one case, a mutation causes both derivatives to sustain an additional 400 loss events per 10^5^ cell divisions. In this case the Ofm index =  (500−100)/(401−1)  = 1. This is the outcome we might expect for a mutation in a kinetochore component, and we would not consider the mutant to be an Ofm mutant. In another case, a mutation causes the 0ORIΔ-ΔR derivative to sustain 10 additional loss events and the 5ORIΔ-ΔR derivative to sustain an additional 900 loss events. In this case the Ofm index  =  (1000−100)/(11−1)  = 90. We would consider this high Ofm index to indicate a specific defect in maintenance of the 5ORIΔ-ΔR fragment.

**Table 2:** Loss rates of chromosome III derivatives in checkpoint mutants (Losses per division ± S. D. × 10^5^).

Strain	5ORIΔ-ΔR	0ORIΔ-ΔR	Ofm index^1^	5ORIΔ	ΔL-6ORIΔ
Wild Type	210±30	3±2	Not defined	9±3	240±40
*sml1Δ*	160±50	18±3	−3	6±2	130±30
*rad9 (ofm14)*	1500±100^2^	19±4^2^	81	39±11	ND
*rad9Δ*	2100±400^2^	32±6^2^	65	30±8	9600±1000
*rad17Δ*	1100±100	12±3	99	ND	ND
*rad24Δ*	980±180	12±3	86	24±5	1500±300
*mec1Δ sml1Δ*	1400±200	33±7	40, 83^3^	260±40	610±100
*mrc1Δ*	1200±200	150±20	7	530±100	460±100
*chk1Δ*	410±50	9±2	33	N.D.	1100±200
*rad53Δ sml1Δ*	490±90	67±11	4, 7^3^	N.D.	1200±200
*rad53Δ chk1Δ sml1Δ*	880±140	39±7	19, 34^3^	N.D.	9000±1000

1 Ofm index  =  (loss_rate_5ORIΔ-ΔR_mutant_−loss_rate_5ORIΔ-ΔR_wild type_)/(loss_rate_0ORIΔ-ΔR_mutant_−loss_rate_0ORIΔ-ΔR_wild type_).

2 Values from [32].

3 Value calculated using *smlΔ* value for 0ORIΔ-ΔR construct.

We first wished to distinguish the roles of the DNA damage and replication stress response pathways in the maintenance of the 5ORIΔ-ΔR derivative. In budding yeast, these pathways are best distinguished by the effects of mutations in the mediators because the pathways share both upstream and downstream components (Figure 1). The DNA-damage-specific mediator, Rad9p, an ortholog of mammalian 53BP1, is phosphorylated by the PIKK Mec1. Hyper-phosphorylated Rad9p binds the effector kinase Rad53p, an ortholog of Chk2, and facilitates both phosphorylation of Rad53p by Mec1p and activation of Rad53p kinase activity by autophosphorylation [40]–[44]. We previously found that both our original *rad9* allele and the *rad9Δ* allele cause Ofm phenotypes, with mutants strains having Ofm indices of 81 and 65, respectively (Table 2 and [32]). These results indicate the DNA damage response pathway contributes to the maintenance of the 5ORIΔ-ΔR derivative.

The corresponding mediator in the replication stress response pathway is Mrc1p, a homolog of mammalian claspin. Mrc1p plays roles in both the replication stress response and normal replication fork progression [45]–[50]. Analysis of the role of *MRC1* in the maintenance of the 5ORIΔ-ΔR derivative was complicated by its location on chromosome III and its dual role in S phase. We constructed both recipient and donor strains carrying the *mrc1Δ* allele; the 5ORIΔ-ΔR *mrc1Δ* and 0ORIΔ-ΔR *mrc1Δ* fragments were then separately transferred into the *mrc1Δ* recipient strain by chromoduction. Both 5ORIΔ-ΔR and 0ORIΔ-ΔR fragments were destabilized in the homozygous *mrc1Δ* strain, resulting in a low Ofm index (Table 2); the *mrc1Δ* strain is not an Ofm mutant. A deletion that removed the C-terminal half of the *MRC1* ORF (the allele included in version 1 of the systematic deletion collection) caused a similar loss rate of the 5ORIΔ-ΔR fragment, but the 0ORIΔ-ΔR loss rate was about 10-fold lower than in the complete ORF deletion strain, suggesting that the N-terminus of Mrc1p may contribute to maintenance of the 0ORIΔ-ΔR fragment (data not shown).

To distinguish between the roles of the replication stress response and fork progression functions of Mrc1p in the maintenance of the 5ORIΔ-ΔR derivative, we made use of a separation of function allele, *mrc1^AQ^*, made by mutating six consensus Mec1p phosphorylation sites [47]; this allele lacks the replication stress response function of *MRC1*, but retains the fork progression function. Plasmids carrying either wild type *MRC1* or *mrc1^AQ^* complemented the high loss rate of the 5ORIΔ-ΔR fragment in the *mrc1Δ* strain (Table 3). These results indicate that it is the loss of the fork progression function of Mrc1p that destabilizes the 5ORIΔ-ΔR fragment, not the loss of replication stress signaling. Therefore, mutations that impair DNA damage signaling, but not replication stress signaling, cause an Ofm phenotype.

**Table 3:** Loss rate of 5ORIΔ-ΔR derivative in *mrc1Δ* strain transformed with plasmids (Losses per division ± S.D. × 10^5^).

no plasmid	pRS416	pmrc1^AQ^	pMRC1
1200±200	1200±200	180±30	230±40

**Figure 4:**
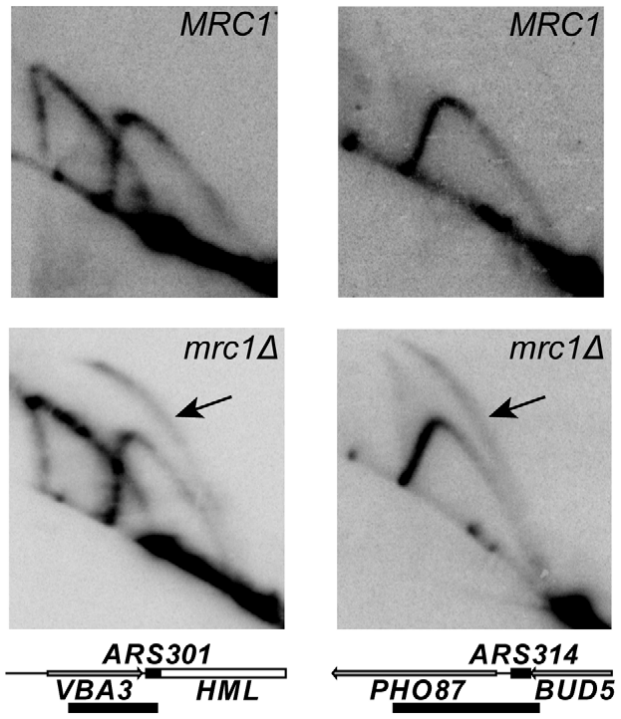
Activity of dormant origins in the *mrc1Δ* mutant. Genomic DNA was prepared from *MRC1* (YDN324) and *mrc1Δ* (YDN337), strains lacking *ARS305*. Southern blots of 2D gels of replicating DNA were probed to detect either *ARS301* (left column) or *ARS314* (right column). The detection of bubble-shaped replication intermediates, indicated by the arrows, demonstrates that both origins are active in the *mrc1Δ* mutant; both origins are inactive in the *MRC1* strain. Diagrams of the 4.8 kb *Nde*I fragment containing *ARS301* and the 3.5 kb *Cla*I-*Eco*RV fragment containing *ARS314* are shown. The black boxes on the map lines indicate the locations of the ARS elements; the bars below the maps indicate the locations of the probes. The *ARS301* probe also hybridized to a 7.1 kb *Nde*I fragment on chromosome XI containing the *VBA5* gene.

We further tested the role of *MRC1* in replication fork progression in our YKN10 background by examining the activation of dormant origins on chromosome III using 2D gel electrophoresis. These origins are inactive in the wild type strain because they are replicated by a fork from an adjacent origin before they can fire. Dormant origins can be activated by deletion of adjacent origins, which causes a delay in the time at which forks from the nearest remaining origins reach them, giving them an opportunity to fire [31], [51]. The dormant origin *ARS304* is also activated in an *mrc1Δ* strain [49] in which forks progress slowly [48], [49]. To explore the generality of this phenomenon, we examined the activation of three dormant origins on chromosome III: *ARS301*, *ARS304* and *ARS314*. As shown in Figure 4, replication initiation at *ARS301* and *ARS314*, revealed by the presence of bubble-shaped intermediates, was detected in the *mrc1Δ* mutant, but not in the *MRC1* strain; *ARS304* was also active in the mutant (data not shown). Thus, activation of dormant origins is a general phenomenon in *mrc1Δ* strains that most likely reflects slow fork progression.

Deletions of other components of the DNA damage and replication stress response pathways also caused Ofm phenotypes. Deletions of genes encoding sensors shared by both pathways, including *RAD17*, which encodes a subunit of a PCNA-like clamp, and *RAD24*, which encodes the large subunit of its clamp loader (see Figure 2), caused Ofm phenotypes with Ofm indexes of 100 and 85, respectively (Table 2). Although it was not scored as a potential Ofm mutant in the primary screen, further examination revealed that deletion of *DDC1*, which encodes another subunit of the clamp, caused colonies of strains carrying the 5ORIΔ-ΔR fragment to sector similarly to the *rad17Δ* strain (Figure S1). Genes encoding other shared sensors were not examined because they are essential, including *RFC2*, *RFC3*, *RFC4*, *RFC5*, *DDC2* and *DPB11* (Figure 1).

Sensors activate PIKKs shared by both pathways. In *S*. *cerevisiae*, the ATR homolog, Mec1p, plays a more important role in the detection and repair of DNA damage than does the ATM homolog, Tel1p [52]. *MEC1* is essential and was not in our screen; however the lethality caused by the *mec1Δ* allele can be suppressed by deletion of the ribonucleotide reductase inhibitor encoded by *SML1* [53]. The *sml1Δ* mutation did not increase the loss rate of the 5ORIΔ-ΔR derivative, though it did slightly elevate the loss rate of the 0ORIΔ-ΔR derivative (Table 2). Since the *sml1Δ* strain is not an Ofm mutant, we examined *mec1Δ* in the *sml1Δ* background. The *mec1Δ* allele confers an Ofm phenotype indicating by its Ofm index of 40 (Table 2). The other PIKK, Tel1p, does not contribute to maintenance of the 5ORIΔ-ΔR fragment. The loss rate of this fragment in the *tel1Δ* mutant was 2.3±0.4×10^−3^ per division, similar to its loss rate in the wild type strain, and its loss rate in the *mec1Δ tel1Δ* double mutant was 1.3±0.2×10^−2^, similar to its loss rate in the *mec1Δ* mutant.

Downstream of the mediator, Rad9p, are the two effector kinases, Chk1p and Rad53p, homologues of the mammalian kinases, Chk1 and Chk2, respectively. The *chk1Δ* strain was not scored as a potential Ofm mutant in the primary screen; however, further examination revealed that this strain had an Ofm phenotype, with an Ofm index of 33 (Table 2). This result implicates Chk1p in transducing the signal from Rad9p to downstream targets. The *rad53Δ* mutant was not in the screen because it is inviable, but its inviability is suppressed by deletion of *SML1*. We found that the *rad53Δ sml1Δ* double mutant did not have an Ofm phenotype (Ofm index  = 7) because the *rad53Δ* mutation caused an increase in the loss rate of the 0ORIΔ-ΔR fragment (Table 2). The increased loss rate of the 0ORIΔ-ΔR fragment in the *rad53* strain indicates that Rad53p contributes to the maintenance of chromosomes with a normal complement of replication origins and is consistent with its well-documented role in response to DNA damage [22]. However, the loss rate of the 5ORIΔ-ΔR fragment was increased about 3-fold relative to the *sml1Δ* control, raising the possibility that Rad53p also contributes to the maintenance of this fragment. We examined the loss rate of the 5ORIΔ-ΔR fragment in a *sml1Δ rad53Δ chk1Δ* strain and found that its loss rate in the triple mutant was 880±140×10^−5^, approximately equal to the sum of the loss rates in the *sml1Δ rad53Δ* and *chk1Δ* mutants and nearly as high as the loss rates in strains carrying deletions of upstream components of the checkpoint pathway (Table 2). The Ofm index of the triple mutant was similar to that of the *chk1* strain. Taken together, these results are consistent with the idea that Chk1p is primarily responsible for transducing the signal from Rad9p to downstream effectors, with Rad53p making a relatively small contribution to the maintenance of the 5ORIΔ-ΔR fragment as long as Chk1p is active, but becoming important in the absence of Chk1p.

### Recombinational repair is not important for maintaining ORIΔ chromosome derivatives

*RAD52* is required for virtually all homology-based double-strand break repair mechanisms, including break-induced replication and single-strand annealing (reviewed by Symington [54]). Our previous work showed that a *rad52* mutant does not have an Ofm phenotype [31]; for this analysis we measured the stabilities of the 5ORIΔ-ΔR and 0ORIΔ-ΔR fragments (Figure 2) in wild type and *rad52* strains in the CF4-16B strain background (Table S2), which differs slightly from the YKN10 background used in experiments summarized in Table 2. The 0ORIΔ-ΔR fragment was lost at a rate of 7×10^−5^ in the wild type strain and 9.5×10^−4^ in the *rad52* strain, while the 5ORIΔ-ΔR fragment was lost at a rate of 1.5×10^−3^ in the wild type strain and 3.1×10^−3^ in the *rad52* strain, leading to an Ofm index of 1.8 [31]. Confirming and extending these results, strains carrying deletions of ten genes in the *RAD52* epistasis group (*RAD50*, *RAD51*, *RAD52*, *RAD54*, *RAD55, RAD57*, *RAD59*, *RDH54*, *MRE11*, and *XRS2*) all showed wild type sectoring in our primary screen (Figure S2). These results indicate that, in otherwise wild type strains, recombinational repair is not required for maintenance of ORIΔ chromosome derivatives.

### Stabilities of chromosome III derivatives with efficient origins and a large inter-origin gap distinguish *mec1Δ* and *mrc1Δ* mutants from *rad9Δ* and *rad24Δ* mutants

By deleting the five efficient origins from the 5ORIΔ-ΔR fragment, we altered both the positions at which replication most likely initiates and the distances that individual replication forks travel. The high loss rates of the 5ORIΔ-ΔR fragment seen in the DNA damage response mutants could result from difficulty in initiating replication, difficulty in replication fork progression, or both. To address this issue, we examined stabilities of two additional derivatives of chromosome III, the full-length 5ORIΔ chromosome and the ΔL-6ORIΔ fragment (Figure 2), in these mutants. The 5ORIΔ-ΔR fragment used in our mutant screen is truncated to the right of the *ARS310* deletion. The full-length 5ORIΔ chromosome carries the same deletions of the five efficient origins as the 5ORIΔ-ΔR fragment, but retains origins distal to the *ARS310* deletion; the inefficient origin, *ARS313*, is located about 20 kb distal to the *ARS310* deletion, and the efficient origin, *ARS315*, is located about 50 kb distal [55]. This derivative is as stable as the 0ORIΔ-ΔR derivative in the wild type strain and the *sml1Δ* mutant. The ΔL-6ORIΔ fragment was derived from the full-length 5ORIΔ chromosome by removing the centromere-associated inefficient origin, *ARS308*, and fragmenting the chromosome to the right of *ARS304*, which removed *ARS304*, the dormant origins associated with *HML* and the left telomere. This derivative is as stable as the 5ORIΔ-ΔR derivative in the wild type strain and the *sml1Δ* mutant (Table 2). In both 5ORIΔ and ΔL-6ORIΔ derivatives, the origin-deleted region to the left of *ARS313* can be replicated by forks that initiate at *ARS313* or at origins further to the right. In 5ORIΔ, but not in ΔL-6ORIΔ, there also exists the potential for the origin-deleted region to be replicated by forks that initiate at one of the normally-dormant *HML*-associated origins.

If a mutant has an initiation defect, then the presence of additional origins on 5ORIΔ and ΔL-6ORIΔ derivatives should suppress the Ofm phenotype. Conversely, if a fork progression defect creates difficulty in completing replication of a large inter-origin gap, the presence of additional origins should not suppress the defect. The ΔL-6ORIΔ fragment provides a particularly stringent test of fork progression and/or fork stability, because a collapsed leftward-moving fork initiated at *ARS313* or *ARS315* cannot be rescued by a fork initiated at one of the HML-associated dormant origins.

**Figure 5:**
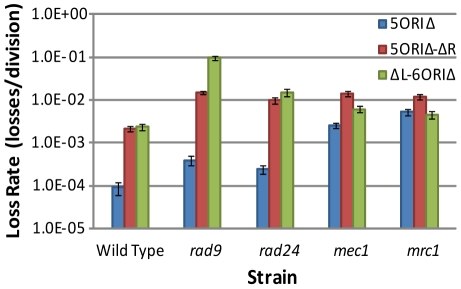
Comparisons of loss rates of ORIΔ derivatives in checkpoint mutants. Selected data from Table 2 are shown. The *mec1* data are from the *mec1Δ sml1Δ* strain.

We first examined the stability of these larger gapped constructs in the *mrc1Δ* mutant because it has a known fork progression defect [48], [49]. *MRC1* was deleted from the full-length 5ORIΔ chromosome to avoid complementation; *MRC1* is distal to *ARS304* so, like the dormant origins, it is absent from the ΔL-6ORIΔ fragment. In *mrc1Δ* mutants, loss rates of the full-length 5ORIΔ chromosome and the ΔL-6ORIΔ fragment were similar, and were about 2.5-fold lower than the loss rate of the 5ORIΔ-ΔR fragment (Figure 5, Table 2). These results are consistent with our expectation that the additional origins on these two derivatives would not suppress the fork progression defect of *mrc1Δ*. Activation of *HML*-associated dormant origins does not appear to contribute to the stability of the full-length 5ORIΔ chromosome in the absence of Mrc1p, because the ΔL-6ORIΔ fragment, which lacks *HML-*associated dormant origins, showed a loss rate similar to 5ORIΔ. The 2.5-fold higher rate of loss of the 5ORIΔ-ΔR fragment likely reflects the fact that replication of this fragment is at least partially dependent upon activation of *HML*-associated dormant origins, and that these origins are less efficient than the origins present on the right arm in the full-length 5ORIΔ chromosome and the ΔL-6ORIΔ fragment (Figure 5).

Consistent with the observation of Cha and Kleckner [56] that Mec1p stabilizes forks in slow replication zones, we found that the *mec1Δ* mutant behaved similarly to the *mrc1Δ* mutant. The 5ORIΔ chromosome was unstable in a *mec1Δ* strain (Figure 5, Table 2), suggesting a fork progression defect. The loss rate of the ΔL-6ORIΔ fragment was less than three-fold higher than that of the full-length 5ORIΔ chromosome, suggesting that the *HML*-associated dormant origins make only a small contribution to the stability of the full-length 5ORIΔ chromosome in the absence of Mec1p.

Results obtained with the *rad9 and rad24Δ* mutants contrasted sharply with the *mrc1Δ* and *mec1Δ* results. The full-length 5ORIΔ chromosome was substantially more stable than 5ORIΔ-ΔR or ΔL-6ORIΔ in the absence of Rad9p or Rad24p, with loss rates about 40-fold lower than the 5ORIΔ-ΔR fragment and only two-fold higher than the 0ORIΔ-ΔR fragment (Table 2 and Figure 5). By contrast, in *mrc1Δ* and *mec1Δ* strains, the full-length 5ORIΔ chromosome is 10- to 20-fold less stable than 0ORIΔ-ΔR.

The relative stability of 5ORIΔ-ΔR in *rad9 and rad24Δ* mutants might indicate that the presence of efficient origins to the right of the origin-deleted region could suppress the Ofm phenotype of these mutants. If this were the case, then the loss rate of the ΔL-6ORIΔ fragment should also be low. However, the loss rates of this fragment were as high as or higher than the 5ORIΔ-ΔR fragment in both mutants. The high loss rates of both the 5ORIΔ-ΔR fragment and the ΔL-6ORIΔ fragment indicate that maintenance of the full-length 5ORIΔ chromosome in *rad9* and *rad24Δ* strains requires the presence of replication origins on both sides of the ORIΔ gap, and suggest that a single fork cannot traverse the gap in these strains.

One explanation for the lower stability of the ΔL-6ORIΔ fragment in the *rad9Δ* strain than in a *mec1Δ sml1Δ* strain is that in the absence of Rad9p, Mec1p kinase activity is deleterious. If this were the case, the loss rate of ΔL-6ORIΔ fragment in a *rad9Δ mec1Δ sml1Δ* triple mutant should be the same as in the *mec1Δ sml1Δ* strain. Alternatively, a second pathway, possibly Tel1p-dependent, could activate Rad9p in the absence of Mec1p, or Rad9p could have a DNA-damage-response-independent function that contributes to the maintenance of the ΔL-6ORIΔ fragment. In both of these cases, the triple mutant should have a loss rate similar to the *rad9Δ* strain. The loss rates of the ΔL-6ORIΔ fragment were 6.9±0.6×10^−2^ in a *rad9 sml1Δ* strain and 5.1±0.4×10^−2^ in a *rad9 mec1Δ sml1Δ* strain, suggesting that a second pathway activates Rad9p. Alternatively Rad9p has a function that is independent of its role in the DNA damage response pathway in maintenance of the ΔL-6ORIΔ fragment (see Discussion).

Finally, the behavior of the ΔL-6ORIΔ fragment in the effector kinase mutants provides strong support for idea that Rad53p becomes important for the maintenance of ORIΔ chromosomes in the absence of Chk1p. The loss rates of the ΔL-6ORIΔ derivative in the *chk1Δ* and *rad53Δ sml1Δ* strains were similar and elevated approximately 2-fold relative to the 5ORIΔ-ΔR derivative. The loss rate in the *chk1Δ rad53Δ sml1Δ* mutant was approximately 10-fold higher and was equal to the very high loss rate seen in the *rad9Δ* mutant (Table 2).

### Activation of dormant origins associated with *HML* in *mec1* and *rad53* strains

**Figure 6:**
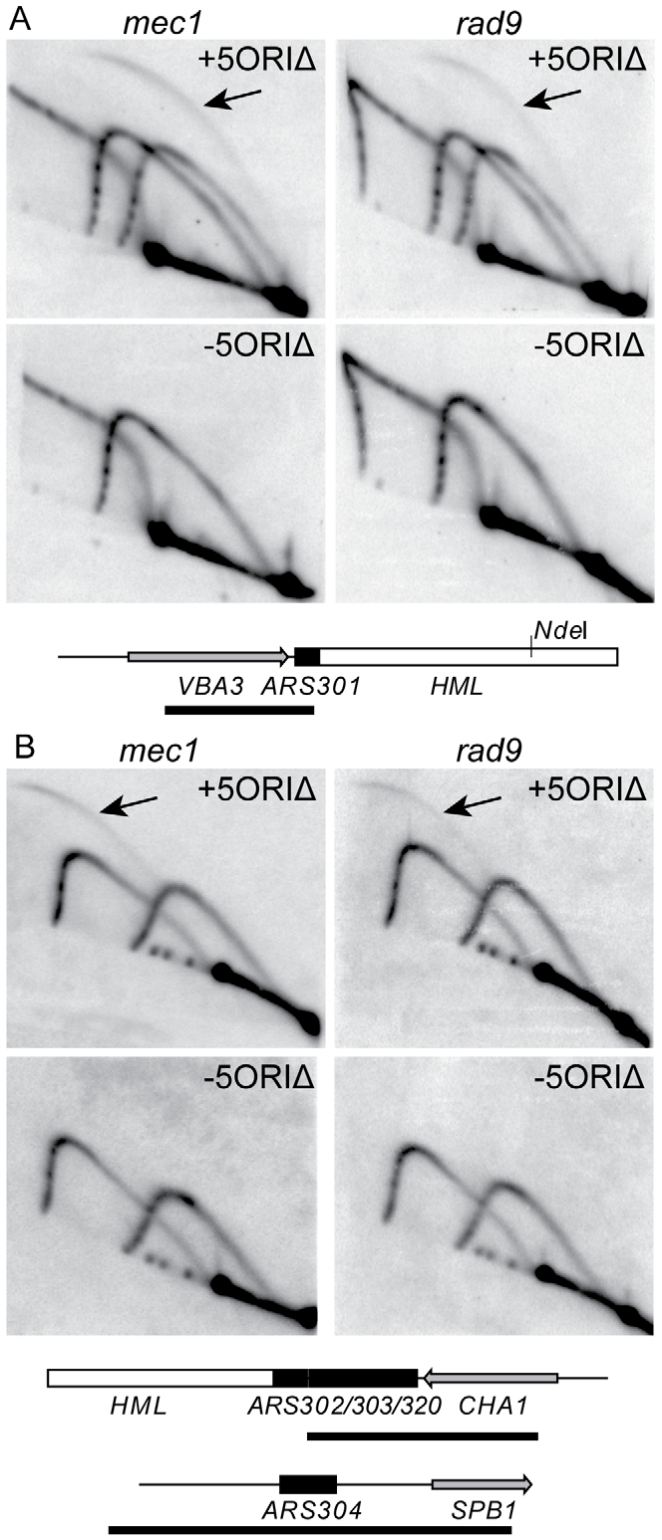
Activity of dormant origins on full-length 5ORIΔ chromosome in *mec1* and *rad9* mutants. Genomic DNA was prepared from *mec1Δ* (YIC110) and *rad9Δ* (YJT135) strains carrying the full-length 5ORIΔ chromosome (+5ORIΔ) and from strains that had lost the 5ORIΔ chromosome (-5ORIΔ). A. Southern blots of 2D gels were probed to detect *ARS301*. Replication intermediates of 4.8-kb *Nde*I fragment from the balancer chromosome and a 4.1-kb *Nde*I fragment from the full-length 5ORIΔ chromosome are shown. The *mec1* gel was run longer in the first dimension than the *rad9* gel. Bubble-shaped replication intermediates, indicated by arrows, arise only from the smaller *Nde*I fragment. Below the blots is a diagram of the *ARS301* fragment as in Figure 4, except that the polymorphic *Nde*I site is indicated. This site is present on the full-length 5ORIΔ chromosome and absent from the balancer chromosome. The bar below the map indicates the probe. The *ARS301* probe also hybridized to a 7.1 kb *Nde*I fragment from chromosome XI containing the *VBA5* gene. B. Southern blots of *Fsp*I+*Sph*I+*Cla*I-cut DNA probed to detect *ARS302*/*ARS303*/*ARS320* and *ARS304* are shown. Bubble-shaped replication intermediates, indicated by the arrows, arise only from *ARS302*/*ARS303*/*ARS320* in the strain carrying the 5ORIΔ chromosome. Diagrams of the 4.5-kb *Fsp*I-*Cla*I fragment containing *ARS302*/*ARS303*/*ARS320* and 3.2-kb *Fsp*I-*Sph*I fragment containing *ARS304* are shown below the blots. ARS elements are indicated by the black boxes, and the bar below each map indicates the probe.

The loss rate of the full-length 5ORIΔ chromosome was much higher in the *mrc1Δ* and *mec1Δ* strains than in the *rad24Δ* and *rad9* strains. It appears that the dormant origins associated with *HML* near the left end of the full-length 5ORIΔ chromosome contribute to the maintenance of this chromosome in wild-type, because derivatives truncated to remove *HML*-associated dormant origins showed higher loss rates than derivatives containing them (Figure 5,Table 2 and [31]). Increased activation of these dormant origins in *rad9* and *rad24Δ*, as compared to in *mrc1Δ* and *mec1Δ*, could explain the differences in stability of the full-length 5ORIΔ chromosome in these two sets of mutants. Therefore, we examined the activation of the dormant origins *ARS301*, *ARS302*/*ARS303*/*ARS320* (three closely-spaced ARS elements), and *ARS304* on the full-length 5ORIΔ fragment by 2D gel analysis (Figure 6). Both bubble- and Y-shaped replication intermediates were detected at *ARS301* in *mec1Δ* and *rad9Δ* strains, indicating that this origin is activated in a subset of the cells in both strains. A fortuitous restriction-site polymorphism allowed us to distinguish the signal arising from the balancer chromosome from that arising from the 5ORIΔ chromosome. Bubble-shaped intermediates were detected only in strains where the 5ORIΔ chromosome was present, indicating that *ARS301* fires only on the 5ORIΔ chromosome. Similarly, we found bubble arcs arising from the *ARS302/ARS303/ARS320* cluster in *mec1Δ* and *rad9Δ* strains, but only when the 5ORIΔ chromosome was present. *ARS304* was not detectably active in either mutant (Figure 6). In all cases, the intensity of the bubble arc was less than that of the Y arc, indicating that in the majority of cells each ARS was passively replicated.

We quantitated the percent of bubble-shaped replication intermediates produced by the 5ORIΔ chromosome, using two approaches to quantitate the signal (Methods and Table S3). *ARS301* initiated replication in 1.7–6.3% of the population in the *rad9Δ* strains, and in 7.4–14.6% of the population in *mec1Δ sml1Δ* strains. The range of values for the *ARS302/ARS303/ARS320* cluster was similar, 2.3–7.6% in *rad9Δ* strain and 9.4–15.7% in the *mec1Δ sml1Δ* strains. Thus the dormant replicators are 2-to 3-fold more active in *mec1Δ* strains than in *rad9Δ* strains, indicating that the higher stability of the 5ORIΔ chromosome in *rad9Δ* strains cannot be explained by increased activation of dormant origins.

The activation of *HML*-associated origins in the *rad9Δ* strain may account for the differences in stability of the 5ORIΔ chromosome and the ΔL-6ORIΔ derivative. The *HML*-associated origins fire only late in S phase [51], [57]. Leftward-moving forks normally reach them before they are programmed to fire. In the *rad9Δ* strain, approximately 10% of cells activate either *ARS301* or the *ARS302/ARS303/ARS320* cluster in the full length 5ORIΔ chromosome. About 10% of *rad9* cells lose the ΔL-6ORIΔ fragment (Table 2), suggesting that about 10% of the forks initiated to the right of the gap fail to traverse the gap in *rad9* mutants. In this situation, the ΔL-6ORIΔ fragment, which lacks the *HML*-associated dormant origins, would be lost as a result of incomplete replication. In contrast, only 0.03% of *rad9* cells lose the 5ORIΔ chromosome (Table 2) because, in the 10% of cells in which leftward-moving forks fail to traverse the gap, firing of one of the *HML*-associated dormant origins allows the replication of this chromosome to be completed.

Unlike the 5ORIΔ-ΔR fragment and the full-length 5ORIΔ chromosome, the ΔL-6ORIΔ fragment was structurally unstable. Stable derivatives that had lost the cloNAT-resistance marker present at left-hand end of the fragment (Figure 2) arose in the *rad9*, *rad24*, and *mec1* mutants. The rates of production of these stable derivatives were similar to the loss rates of the ΔL-6ORIΔ fragment measured in these strains (Table S4). Twelve stable derivatives of the ΔL-6ORIΔ fragment produced by the *rad9* strain migrated on pulsed-field gels with the full-length balancer chromosome, suggesting that chromosome III sequences distal to the fragmentation point had been restored (data not shown). One possible mechanism for the production of these stable derivatives is that replication forks collapse and are processed into double-strand breaks that are repaired by break-induced replication [58] using the balancer chromosome as a template.

## Discussion

We employed a novel modification of the SGA method to screen for mutations that preferentially destabilize a chromosome III derivative lacking efficient replication origins. The modification utilized a single chromosome transfer technique, chromoduction, to transfer the 5ORIΔ-ΔR fragment into an ordered array of the viable ORF deletion collection. Yuen *et al*. [59] carried out similar colony-sectoring screens of the viable deletion collection using two chromosome fragments. Of the 66 chromosome transmission fidelity (*ctf*) mutants identified in these screens, 14 were also identified in our screen. As expected, given that the *ctf* mutants were identified using chromosome fragments carrying a normal complement of replication origins, the majority of the *ctf* mutants we re-identified were found in the non-Ofm or possible/probable Ofm classes. The two scored as Ofm mutants are *ctf18Δ* and *mad2*. It seems likely that many *ctf* mutants were not identified in our screen because they caused only small increases in the rate of loss of the 5ORIΔ-ΔR fragment. Approximately 60% of the loss rates measured for chromosome fragments in *ctf* mutants are less than or equal to the loss rate of the 5ORIΔ-ΔR fragment; increases of that magnitude would not have been detected in our visual screen.

### Role of the DNA damage response pathway in the maintenance of ORIΔ chromosome derivatives

Our results indicate that the DNA damage signaling pathway, but not the replication stress signaling pathway, contributes to the maintenance of the 5ORIΔ-ΔR fragment. While the DNA damage and replication stress response pathways share many components (Figure 1), mutation of the DNA-damage-tocheckpoint-signaling mediator, Rad9p, preferentially destabilized the 5ORIΔ-ΔR fragment, but a checkpoint-deficient mutation in the replication-stress-specific signaling mediator, Mrc1p, did not. Mutations in many of the shared signaling components also caused Ofm phenotypes.

We found unexpected differences in the contributions that the DNA damage signaling pathway makes to maintenance of ORIΔ chromosome derivatives and the contributions that it makes to DNA damage resistance. First, the DNA damage signaling pathway detects and stabilizes forks stalled at sites of damage and facilitates repair or bypass of the damage; studies with DNA damaging agents [60]–[63] indicate that this function is more strongly dependent on *RAD53* than on *CHK1*. Based on the results presented here, the DNA damage signaling pathway also contributes to the replication of large inter-origin gaps, which can arise when several adjacent origins fail to fire. Such gaps appear commonly during the replication of the rDNA array [14]. The 5ORIΔ-ΔR fragment, the full-length 5ORIΔ chromosome and the ΔL-6ORIΔ fragment mimic these large gaps, and the pathways identified by the Ofm mutants may have arisen to facilitate the replication of large inter-origin gaps. Interestingly, this function appears to be facilitated primarily by *CHK1* with a contribution from *RAD53* evident in the absence of *CHK1*.

Second, we found that *mec1Δ* and *mrc1Δ* mutations have different effects than *rad9Δ* and *rad24Δ* mutations on the stabilities of the ΔL-6ORIΔ and full-length 5ORIΔ derivatives. Dormant origins near the left end of chromosome III are more strongly activated in a *mec1Δ* mutant than in a *rad9* mutant (Figure 6), suggesting that in the *mec1Δ* strain the *HML*-associated dormant origins have more time to fire. However, removing the dormant origins, as in the ΔL-6ORIΔ fragment, caused a 16-fold greater increase in the rate of chromosome loss in the *rad9* strain than in the *mec1Δ* strain (Table 2), suggesting that forks fail to reach the left end more often in the *rad9* strain. One explanation for this disparity is that an alternative pathway activates Rad9p in *mec1Δ* cells, which results in stabilization of replication forks and allows them to progress, albeit slowly, in the absence of Mec1p [56]. In *mec1* mutants, we suggest that slow fork progression through the long *ARS305Δ – ARS310Δ* gap allows time for the activation of either *ARS301* or the *ARS302/ARS303/ARS320* cluster in ∼20% of the cells (Figure 6). However in the absence of the dormant origins, as in the ΔL-6ORIΔ fragment, these slow-moving forks are able to complete replication through the gap to the telomere in >99% of the cells (Table 2). Tel1p, which is also a PIKK, is a candidate for activation of Rad9p, in this situation. However, our observation that Tel1p did not contribute to the stability of the 5ORIΔ-ΔR fragment in either the presence or absence of Mec1p (see Results) suggests that Tel1p does not contribute to this pathway. In the absence of Rad9p, we suggest that forks initiated to the right of the *ARS305Δ – ARS310Δ* gap simply fail to traverse the gap approximately 10% of the time (Table 2), and that, in the absence of the dormant origins, replication of the chromosome is not completed, leading to segregation of the partially replicated molecule and chromosome loss. An alternative explanation for the disparity is that Rad9p has a function that is independent of its role in the DNA damage response pathway.

Finally, we found that strains carrying deletions of ten genes in the *RAD52* epistasis group did not show elevated loss rates of the 5ORIΔ-ΔR fragment. Since genes in this epistasis group are required for all homology-dependent repair processes, including double-strand break repair, break-induced replication and replication fork restart, these results suggest that replication of this ORIΔ derivative does not require repair of DNA damage or double-strand breaks.

Our favored model for the role of the DNA damage response pathway in the replication of ORIΔ chromosome derivatives is based on the idea that replication forks age, i.e. that the probability of fork arrest due to failure of a replisome component increases with the distance the fork has traveled. We refer to these forks as crippled, to distinguish them from forks that are stalled (arrested by DNA damage or nucleotide depletion with replisome intact) or collapsed (replisome disassembled), and to reflect the need for some replisome component to be replaced or modified in order to continue elongation. These crippled forks are then recognized and restored by a *RAD9*-and *CHK1*-dependent pathway. The restart of these crippled forks is independent of homologous recombination because there is no DNA damage to be bypassed, and, therefore, double-strand breaks are therefore not formed. If a fork were arrested due to failure of a replisome component, there would be no impediment to elongation once the replisome is reconstituted.

There are alternative models to explain the role of the DNA damage response pathway in maintaining the 5ORIΔ-ΔR fragment, which has a large inter-origin gap. The simplest is that the DNA damage response monitors the completion of replication. However, the evidence for such a checkpoint is not compelling (see Introduction). Debate over the existence of a replication completion checkpoint is ongoing; our observations provide only circumstantial evidence in favor of such a checkpoint.

Another model to explain the role of the DNA damage response pathway in maintaining fragments with large inter-origin gaps suggests that forks stall at sites of endogenous DNA damage and are stabilized by this pathway. The 5ORIΔ-ΔR and ΔL-6ORIΔ fragments would be especially sensitive to such events in the absence of the DNA damage response because the stalled forks would collapse. In the case of the 0ORIΔ-ΔR fragment, which has a full complement of replication origins, a collapsed fork could be rescued by a converging fork from an adjacent origin. In contrast, the 5ORIΔ-ΔR fragment has fewer initiation events, so a collapsed fork would be rescued less often by a converging fork, resulting in an elevated loss rate in a DNA damage checkpoint mutant. Consistent with this suggestion, our analysis of individual 5ORIΔ-ΔR molecules in wild type cells suggests that replication initiates at only one or two places per molecule, but at different places on different molecules (Wang et al., manuscript in preparation).

The enhanced stability of the full-length 5ORIΔ chromosome compared to the ΔL-6ORIΔ fragment in the *rad9* and *rad24Δ* mutants is also consistent with this endogenous damage model, as a collapsed leftward-moving fork in the 5ORIΔ chromosome can be rescued by a fork initiating at one of the dormant origins near *HML*. Our finding that *mec1Δ* confers an Ofm phenotype while *tel1Δ* does not is also consistent with this model because *MEC1* plays a more important role in the tolerance of DNA damage than does *TEL1* [52].

However, this endogenous damage model is challenged by findings that fork stabilization at sites of DNA damage and survival are more strongly dependent on *RAD53* than on *CHK1* [60]–[66], whereas *CHK1* makes a more important contribution than *RAD53* to 5ORIΔ-ΔR fragment maintenance, suggesting that the DNA damage response is not simply stabilizing forks in response to damage. While Segurado and Diffley [61] have suggested a role for *CHK1* in stabilizing replication forks, that function was detected only in the absence of both *RAD53* and *EXO1*, which encodes a nuclease responsible for fork collapse in the absence of *RAD53*. Thus, it seems unlikely that this explains the contribution of *CHK1* to 5ORIΔ-ΔR fragment maintenance. Another problem is that deletions of genes, whose products are required for mismatch repair, repair of UV damage, and homologous recombination, did not increase the loss rate of the 5ORIΔ-ΔR fragment in the primary screen, as would have been expected if DNA damage-provoked fork collapse was responsible for loss of this fragment.

Replication fork aging also suggests an explanation for the close spacing of replication origins in *S. cerevisiae*. A median inter-origin distance of 36 kb was estimated from visualization of replicating molecules by electron microscopy (reviewed by Newlon [67]), and a similar median distance, 34 kb, was estimated using the genome-wide replication timing data of Raghuraman *et al*. [4]. Based on a median fork rate of 2.3 kb per minute and an S phase of 55 minutes [4], a single fork from the earliest firing origin would be able to replicate ∼120 kb and a fork from an origin activated in the middle of S phase would be able to replicate ∼60 kb. Thus, origins are spaced more closely than predicted by the median origin activation time and rate of fork movement. The observed high density of origins may insure that gaps too long to be reliably replicated do not occur, even if several adjacent origins fail to fire.

### DNA-replication-linked genes

Pan et al. described a DNA Integrity Network of 78 genes on the basis of synthetic fitness or lethality defects [68]. Sixteen of these genes are believed to have roles in S phase checkpoints. Deletions of eight of these genes cause an Ofm phenotype: *RAD9*, *RAD17*, *RAD24*, *CTF18*, *MEC1*, *DDC1*, *CHK1*, and *RAD53*. Deletions of two other genes in this group, *csm3Δ* and *tof1Δ,* were scored possible Ofm mutants.

In addition to the checkpoint genes, our Ofm mutants included deletions of two other genes from the DNA Integrity Network, *HST3* and *POL32*, both of which have links to DNA replication. *HST3* encodes a NAD^+^-dependent histone H3 lysine-56 deacetylase [69]–[71]. Our analysis of *hst3* mutants will be presented elsewhere; it indicates that the Ofm phenotype of *hst3Δ* results from a fork progression defect (Irene et al. manuscript submitted). *pol32Δ* mutants, which lack a nonessential subunit of DNA polymerase Δ, also show fork progression defects, which may explain their Ofm phenotype [72]–[75].

In summary, we have identified a set of genes whose products facilitate replication of large inter-origin gaps. This set is enriched in components of the DNA damage and replication stress signaling pathways. Replication of large inter-origin gaps shows several surprising features: Dependence on the DNA-damage-specific mediator, Rad9p, rather than the replication-stress-specific mediator, Mrc1p; a stronger dependence on the effector kinase, Chk1p than Rad53p, and no dependence on homologous recombination

## Methods

### Strains and media

Yeast strains are listed in Table S2. All strains are isogenic with YPH499 [76], except the full-length and fragmented chromosome donor strains, which are in the CF4-16B background [31], and YJT242 (and its parent Y7029) and the viable ORF deletion collection, which are related to S288C [77]. SGA selection media were prepared as described in [78]. Chromoductants for the SGA screen were selected on -Ade -Leu -Lys -Arg dropout plates containing 60 µg/ml canavanine (Sigma) and 10 µg/ml thialysine (Sigma). Chromoductants in the YKN10 background were selected on -Leu-Trp -Arg dropout plates containing 60 µg/ml canavanine and 10 µg/ml cycloheximide (Sigma), except that chromoductants of the ΔL-6ORIΔ fragment were selected on -Leu -Ade -Arg dropout plates containing 100 µg/ml CloNAT (Werner Bioagents, Germany), 60 µg/ml canavanine, and 10 µg/ml cycloheximide. Limiting adenine medium was purchased from US Biologicals.

YJT242 was created by transforming Y7029 with a PCR product carrying the *natMX* gene, amplified from pAG25 [79], flanked by homology to the *ADE2* locus; sequences of primers are available upon request. Individual G418-resistant knockouts were moved into the YKN10 background by transformation with a PCR product amplified from the appropriate strain from the ORF deletion collection (Open Biosystems) using the locus specific A and D primers (www-sequence.stanford.edu/group/yeast_deletion_project/Deletion_primers_PCR_sizes.txt). The *mrc1Δ::NAT* allele was introduced into the YKN10 background using primers and a template generously provided by K. Sugimoto (UMDNJ). This allele was converted to *mrc1Δ::KAN* by transforming YJT294 with *Not*I-cut pFA-KanMX4 [80] and selecting for G418-resistance yielding YJT551. The *his3-Δ367* alleles were generated by fusion PCR and introduced by two-step gene replacement [81]. Primers are available upon request. The *bar1-Δ1327* allele carries a *Bgl*II-*Bsr*GI deletion that removes 1327 bp within the open reading frame.

### SGA screen

In our version of the screen, a strain carrying an *ade2Δ::natMX* mutation, which causes the accumulation of a red pigment in colonies grown on limiting adenine and confers nourseothricin resistance, was mated to the array of viable deletion mutants, each marked with *kanMX*, which confers G418 resistance. The resulting diploids were then sporulated, and double mutant *ade2Δ::natR xxxΔ::kanR* haploid *MAT* ***a*** progeny were selected. The array of double-mutant strains was mated to F510αA1–4, the donor strain, carrying the 5ORIΔ-ΔR derivative of chromosome III marked with *ADE2* (Figure 2). Because the donor strain carries the *kar1-Δ15* mutation, normal karyogamy is inhibited, resulting in inefficient production of diploid cells [82]. During the transient heterokaryon stage, single chromosomes are transferred at low frequency between the two nuclei, a process called chromoduction [35]. The strains were marked to allow selection for rare chromoduction events in which the 5ORIΔ-ΔR fragment was transferred into the *ade2Δ::natR xxxΔ::kanR* nucleus. The 5ORIΔ-ΔR fragment carries *LEU2* at its endogenous locus and an ectopic copy of *ADE2* inserted near the *ARS307* deletion (Figure 2). The corresponding donor strain carrying the 5ORIΔ-ΔR fragment is Leu^+^ and Ade^+^, but canavanine-sensitive and thialysine-sensitive because it carries the wild type *CAN1* and *LYP1* alleles. The double mutant (*ade2Δ::natR xxxΔ::kanR*) strains generated by SGA analysis are Leu^−^, Ade^−^, canavanine-resistant, and thialysine-resistant. Any diploids that form between the donor strain and the *ade2Δ::natR xxxΔ::kanR* double mutant strains are Leu^+^ and Ade^+^, but canavanine-sensitive and thialysine-sensitive because the *can1Δ* and *lyp1Δ* mutations are recessive. The desired chromoduction event results in cells that are Leu^+^ and Ade^+^, because they carry the 5ORIΔ-ΔR fragment, and canavanine- and thialysine-resistant, because they carry the *can1Δ* and *lyp1Δ* mutations. Medium lacking leucine, adenine, arginine, and lysine and containing both canavanine and thialysine selects for these cells. A preliminary screen using approximately 100 strains selected from the viable deletion collection was carried out to determine conditions for the chromoduction. We found that pinning the array of double mutants at the density found in a standard 384 well plate was necessary to ensure efficient mating of the donor strain to the array.

The screen was done in duplicate, and chromoductants from the duplicate arrays were streaked side-by-side on a single plate with limiting adenine for scoring sectoring patterns (see Table S1). This process was completed in less than three months by eight individuals, demonstrating the feasibility of including a chromoduction step in the SGA procedure to transfer a single chromosome or plasmid into the double mutant array. If the phenotype of chromoductants could be scored directly on selective medium, then the entire procedure could be accomplished with robots.

### Loss rate measurements

Chromosome loss rates were determined by fluctuation analysis using the colony isolation method [83]. Red colonies were tested for leucine and tryptophan auxotrophies to distinguish chromosome losses from gene conversions or mitotic recombination events; leucine auxotrophy and nourseothricin-resistance were used in fluctuations involving the ΔL-6ORIΔ fragment. The presence of origin deletions was confirmed by PCR. Loss rates were calculated using the method of Lea and Coulson [84].

### Analysis of replication intermediates

Genomic DNA was prepared from log-phase cultures as described [85], digested with either *Nde*I, *Cla*I+*Eco*RV, or *Fsp*I+*SphI*+*Cla*I, subjected to BND-cellulose (Sigma) chromatography, electrophoresed on neutral-neutral 2D gels, blotted, and hybridized as described [86]. The probe for *ARS301* was the1.3-kb *Eco*RI-*Xho*I fragment from p78_4.6; the probe for *ARS302*/*ARS303*/*ARS320* was the 1.9-kb *Eco*RI-*Hin*dIII fragment from p78_5.2; the probe for *ARS304* was the 3.5-kb *Psh*AI-*Bam*HI fragment from D10B; the probe for *ARS314* was the 1.8-kb *Hin*dIII fragment from pH 1.8 [55], [87]. These fragments were labeled with [α-^32^P] dATP (Perkin Elmer) using the Megaprime DNA-labeling system (GE Healthcare). Images were acquired on a Molecular Dynamics Typhoon 9410, and the exposure was adjusted using ImageQuant 5.2 software. Quantitations of bubble-shaped and Y-shaped replication intermediates were determined using the polygon tool and the line tool of ImageQuant 5.2.

### Photography

Colonies were photographed after ∼5 days of growth at 30°C on limiting adenine plates. Images were acquired as TIFF files with a Nikon D-100 camera fitted with an AF Micro-Nikkor 60 mm f/2.8 D lens. Images were cropped and adjusted for color balance and brightness/contrast in Photoshop.cs v8.0 (Adobe Systems).

## Supporting Information

Figure S1 Sectoring patterns of *aro7Δ*, *ddc1Δ*, and *rad17Δ* strains. The 5ORIΔ-ΔR fragment was introduced into *aro7Δ*, *ddc1Δ* and *rad17Δ* strains by chromoduction, and the chromoductants were streaked on plates with limiting adenine and photographed after growth for 5 days.
(1.70 MB TIF)

Figure S2 Sectoring patterns of mutants in the *rad52* epistasis group. Top panels: 5ORIΔ-ΔR chromoductants of *rad50Δ*, *rad51Δ*, *rad52Δ*, *rad54Δ*, *rad55Δ*, *rad57Δ*, *rad59Δ*, *rdh54Δ*, *mre11Δ* and *xrs2Δ* strains isolated in the whole genome screen were streaked on plates with limiting adenine and photographed after growth for 5 days. *aro7Δ* and *rad9Δ* chromoductants were included as controls. Lower panels: Chromoductants were tested for sensitivity to phleomycin, which induces double-stranded breaks, to confirm that the strains carried the expected deletions. Cultures of the strains shown in the top panels were grown overnight in YEPD, serially diluted and spotted on YEPD plates (control) and plates with 0.1 and 1.0 µg/ml phleomycin. YEPD plates and 0.1 µg/ml phleomycin plates photographed after 3 days, 1.0 g/ml phleomycin plates after 5 days. Each of the strains, with the exception of *rad59Δ* and *rdh54Δ*, showed sensitivity, indicating that the sensitive strains carried the expected deletions. *rad59* mutants have been reported to be 10,000-fold less sensitive to gamma irradiation than *rad52* mutants [Bai et al], so the lack of sensitivity of the strain we tested was expected. The sensitivity of *rdh54Δ* to gamma irradiation had not been previously tested but it had been shown not to be sensitive to HO-induced double strand breaks [Klein et al]; we found that an authentic *rdh54Δ* mutant was also not sensitive to phleomycin. [Bai Y, Symington LS (1996) A Rad52 homolog is required for *RAD51*-independent mitotic recombination in *Saccharomyces cerevisiae*. Genes Dev 10: 2025–2037.] [Klein HL (1997) *RDH54*, a *RAD54* homolog in *Saccharomyces cerevisiae*, is required for mitotic diploid-specific recombination and repair and for meiosis. Genetics 147: 1533–1543.]
(6.45 MB TIF)

Table S1 Results of secondary screen for Ofm mutants.
(0.73 MB PDF)

Table S2 Strains used in this study.
(0.06 MB DOC)

Table S3 Quantitation of 2D gels.
(0.03 MB DOC)

Table S4 Appearance of rarely-sectored colonies in strains carrying the ΔL-6ORIΔ fragment.
(0.03 MB DOC)

## References

[1] Bell SP, Dutta A (2002). DNA replication in eukaryotic cells.. Annu Rev Biochem.

[2] Newlon CS, Burke WG, Alberts B, Fox CF (1980). Replication of small chromosomal DNAs in yeast.. Mechanistic Studies of DNA Replication and Recombination.

[3] Feng W, Collingwood D, Boeck ME, Fox LA, Alvino GM (2006). Genomic mapping of single-stranded DNA in hydroxyurea-challenged yeasts identifies origins of replication.. Nat Cell Biol.

[4] Raghuraman MK, Winzeler EA, Collingwood D, Hunt S, Wodicka L (2001). Replication dynamics of the yeast genome.. Science.

[5] Patel PK, Arcangioli B, Baker SP, Bensimon A, Rhind N (2006). DNA replication origins fire stochastically in fission yeast.. Mol Biol Cell.

[6] Donaldson AD, Raghuraman MK, Friedman KL, Cross FR, Brewer BJ (1998). *CLB5*-dependent activation of late replication origins in *S. cerevisiae*.. Mol Cell.

[7] Ferguson BM, Brewer BJ, Fangman WL (1991). Temporal control of DNA replication in yeast.. Cold Spring Harbor Symp Quant Biol.

[8] Friedman KL, Brewer BJ, Fangman WL (1997). Replication profile of *Saccharomyces cerevisiae* chromosome VI.. Genes Cells.

[9] McCarroll RM, Fangman WL (1988). Time of replication of yeast centromeres and telomeres.. Cell.

[10] Reynolds AE, McCarroll RM, Newlon CS, Fangman WL (1989). Time of replication of ARS elements along yeast chromosome III.. Mol Cell Biol.

[11] McCune HJ, Danielson LS, Alvino GM, Collingwood D, Delrow JJ (2008). The temporal program of chromosome replication: genomewide replication in *clb5Δ Saccharomyces cerevisiae*.. Genetics.

[12] Yabuki N, Terashima H, Kitada K (2002). Mapping of early firing origins on a replication profile of budding yeast.. Genes Cells.

[13] Czajkowsky DM, Liu J, Hamlin JL, Shao Z (2008). DNA combing reveals intrinsic temporal disorder in the replication of yeast chromosome VI.. J Mol Biol.

[14] Pasero P, Bensimon A, Schwob E (2002). Single-molecule analysis reveals clustering and epigenetic regulation of replication origins at the yeast rDNA locus.. Genes Dev.

[15] Segurado M, de Luis A, Antequera F (2003). Genome-wide distribution of DNA replication origins at A+T-rich islands in *Schizosaccharomyces pombe*.. EMBO Rep.

[16] Heichinger C, Penkett CJ, Bahler J, Nurse P (2006). Genome-wide characterization of fission yeast DNA replication origins.. EMBO Journal.

[17] Hayashi M, Katou Y, Itoh T, Tazumi A, Yamada Y (2007). Genome-wide localization of pre-RC sites and identification of replication origins in fission yeast.. EMBO Journal.

[18] Dai J, Chuang RY, Kelly TJ (2005). DNA replication origins in the *Schizosaccharomyces pombe* genome.. Proc Natl Acad Sci U S A.

[19] Hyrien O, Marheineke K, Goldar A (2003). Paradoxes of eukaryotic DNA replication: MCM proteins and the random completion problem.. Bioessays.

[20] Lygeros J, Koutroumpas K, Dimopoulos S, Legouras I, Kouretas P (2008). Stochastic hybrid modeling of DNA replication across a complete genome.. Proc Natl Acad Sci U S A.

[21] Branzei D, Foiani M (2005). The DNA damage response during DNA replication.. Curr Opin Cell Biol.

[22] Branzei D, Foiani M (2009). The checkpoint response to replication stress.. DNA Repair (Amst).

[23] Gibson DG, Aparicio JG, Hu F, Aparicio OM (2004). Diminished S-phase cyclin-dependent kinase function elicits vital Rad53-dependent checkpoint responses in *Saccharomyces cerevisiae*.. Mol Cell Biol.

[24] Gibson DG, Bell SP, Aparicio OM (2006). Cell cycle execution point analysis of ORC function and characterization of the checkpoint response to ORC inactivation in *Saccharomyces cerevisiae*.. Genes Cells.

[25] van Brabant AJ, Buchanan CD, Charboneau E, Fangman WL, Brewer BJ (2001). An origin-deficient yeast artificial chromosome triggers a cell cycle checkpoint.. Mol Cell.

[26] Piatti S, Lengauer C, Nasmyth K (1995). Cdc6 is an unstable protein whose de novo synthesis in G1 is important for the onset of S phase and for preventing a ‘reductional’ anaphase in the budding yeast *Saccharomyces cerevisiae*.. EMBO J.

[27] Kelly TJ, Martin GS, Forsburg SL, Stephen RJ, Russo A (1993). The fission yeast *cdc18+* gene product couples S phase to START and mitosis.. Cell.

[28] Pasero P, Duncker BP, Schwob E, Gasser SM (1999). A role for the Cdc7 kinase regulatory subunit Dbf4p in the formation of initiation-competent origins of replication.. Genes Dev.

[29] Lengronne A, Schwob E (2002). The yeast CDK inhibitor Sic1 prevents genomic instability by promoting replication origin licensing in late G(1).. Molecular Cell.

[30] Torres-Rosell J, De Piccoli G, Cordon-Preciado V, Farmer S, Jarmuz A (2007). Anaphase onset before complete DNA replication with intact checkpoint responses.. Science.

[31] Dershowitz A, Snyder M, Sbia M, Skurnick JH, Ong LY (2007). Linear derivatives of *Saccharomyces cerevisiae* chromosome III can be maintained in the absence of autonomously replicating sequence elements.. Mol Cell Biol.

[32] Theis JF, Dershowitz A, Irene C, Maciariello C, Tobin ML (2007). Identification of mutations that decrease the stability of a fragment of *Saccharomyces cerevisiae* chromosome III lacking efficient replicators.. Genetics.

[33] Tong AH, Evangelista M, Parsons AB, Xu H, Bader GD (2001). Systematic genetic analysis with ordered arrays of yeast deletion mutants.. Science.

[34] Tong AH, Boone C (2006). Synthetic genetic array analysis in *Saccharomyces cerevisiae*.. Methods Mol Biol.

[35] Ji H, Moore DP, Blomberg MA, Braiterman LT, Voytas DF (1993). Hotspots for unselected Ty1 transposition events on yeast chromosome III are near tRNA genes and LTR sequences.. Cell.

[36] Garber PM, Rine J (2002). Overlapping roles of the spindle assembly and DNA damage checkpoints in the cell-cycle response to altered chromosomes in *Saccharomyces cerevisiae*.. Genetics.

[37] Kim EM, Burke DJ (2008). DNA damage activates the SAC in an ATM/ATR-dependent manner, independently of the kinetochore.. PLoS Genet.

[38] Maringele L, Lydall D (2002). *EXO1*-dependent single-stranded DNA at telomeres activates subsets of DNA damage and spindle checkpoint pathways in budding yeast *yku70Δ* mutants.. Genes Dev.

[39] Wang Y, Hu F, Elledge SJ (2000). The Bfa1/Bub2 GAP complex comprises a universal checkpoint required to prevent mitotic exit.. Curr Biol.

[40] Beckwith WH, Sun Q, Bosso R, Gerik KJ, Burgers PM (1998). Destabilized PCNA trimers suppress defective Rfc1 proteins in vivo and in vitro.. Biochemistry.

[41] Emili A (1998). MEC1-dependent phosphorylation of Rad9p in response to DNA damage.. Mol Cell.

[42] Feijoo C, Hall-Jackson C, Wu R, Jenkins D, Leitch J (2001). Activation of mammalian Chk1 during DNA replication arrest: a role for Chk1 in the intra-S phase checkpoint monitoring replication origin firing.. J Cell Biol.

[43] Schwartz MF, Duong JK, Sun Z, Morrow JS, Pradhan D (2002). Rad9 phosphorylation sites couple Rad53 to the *Saccharomyces cerevisiae* DNA damage checkpoint.. Mol Cell.

[44] Vialard JE, Gilbert CS, Green CM, Lowndes NF (1998). The budding yeast Rad9 checkpoint protein is subjected to Mec1/Tel1-dependent hyperphosphorylation and interacts with Rad53 after DNA damage.. EMBO J.

[45] Alcasabas AA, Osborn AJ, Bachant J, Hu F, Werler PJ (2001). Mrc1 transduces signals of DNA replication stress to activate Rad53.. Nat Cell Biol.

[46] Katou Y, Kanoh Y, Bando M, Noguchi H, Tanaka H (2003). S-phase checkpoint proteins Tof1 and Mrc1 form a stable replication-pausing complex.. Nature.

[47] Osborn AJ, Elledge SJ (2003). Mrc1 is a replication fork component whose phosphorylation in response to DNA replication stress activates Rad53.. Genes Dev.

[48] Tourriere H, Versini G, Cordon-Preciado V, Alabert C, Pasero P (2005). Mrc1 and Tof1 promote replication fork progression and recovery independently of Rad53.. Mol Cell.

[49] Szyjka SJ, Viggiani CJ, Aparicio OM (2005). Mrc1 is required for normal progression of replication forks throughout chromatin in *S. cerevisiae*.. Mol Cell.

[50] Hodgson B, Calzada A, Labib K (2007). Mrc1 and Tof1 regulate DNA replication forks in different ways during normal S phase.. Mol Biol Cell.

[51] Vujcic M, Miller CA, Kowalski D (1999). Activation of silent replication origins at autonomously replicating sequence elements near the *HML* locus in budding yeast.. Mol Cell Biol.

[52] Sanchez Y, Desany BA, Jones WJ, Liu Q, Wang B (1996). Regulation of RAD53 by the ATM-like kinases MEC1 and TEL1 in yeast cell cycle checkpoint pathways.. Science.

[53] Zhao X, Muller EG, Rothstein R (1998). A suppressor of two essential checkpoint genes identifies a novel protein that negatively affects dNTP pools.. Mol Cell.

[54] Symington LS (2002). Role of *RAD52* epistasis group genes in homologous recombination and double-strand break repair.. Microbiol Mol Biol Rev.

[55] Poloumienko A, Dershowitz A, De J, Newlon CS (2001). Completion of replication map of *Saccharomyces cerevisiae* chromosome III.. Mol Biol Cell.

[56] Cha RS, Kleckner N (2002). ATR homolog Mec1 promotes fork progression, thus averting breaks in replication slow zones.. Science.

[57] Santocanale C, Sharma K, Diffley JF (1999). Activation of dormant origins of DNA replication in budding yeast.. Genes Dev.

[58] Llorente B, Smith CE, Symington LS (2008). Break-induced replication: what is it and what is it for?. Cell Cycle.

[59] Yuen KW, Warren CD, Chen O, Kwok T, Hieter P (2007). Systematic genome instability screens in yeast and their potential relevance to cancer.. Proc Natl Acad Sci U S A.

[60] Sanchez Y, Bachant J, Wang H, Hu F, Liu D (1999). Control of the DNA damage checkpoint by chk1 and rad53 protein kinases through distinct mechanisms.. Science.

[61] Segurado M, Diffley JF (2008). Separate roles for the DNA damage checkpoint protein kinases in stabilizing DNA replication forks.. Genes Dev.

[62] Tercero JA, Longhese MP, Diffley JF (2003). A central role for DNA replication forks in checkpoint activation and response.. Mol Cell.

[63] Lopes M, Cotta-Ramusino C, Liberi G, Foiani M (2003). Branch migrating sister chromatid junctions form at replication origins through Rad51/Rad52-independent mechanisms.. Mol Cell.

[64] Caldwell JM, Chen Y, Schollaert KL, Theis JF, Babcock GF (2008). Orchestration of the S-phase and DNA damage checkpoint pathways by replication forks from early origins.. J Cell Biol.

[65] Lopes M, Cotta-Ramusino C, Pellicioli A, Liberi G, Plevani P (2001). The DNA replication checkpoint response stabilizes stalled replication forks.. Nature.

[66] Sogo JM, Lopes M, Foiani M (2002). Fork reversal and ssDNA accumulation at stalled replication forks owing to checkpoint defects.. Science.

[67] Newlon CS (1988). Yeast chromosome replication and segregation.. Microbiological Reviews.

[68] Pan X, Ye P, Yuan DS, Wang X, Bader JS (2006). A DNA integrity network in the yeast *Saccharomyces cerevisiae*.. Cell.

[69] Celic I, Masumoto H, Griffith WP, Meluh P, Cotter RJ (2006). The sirtuins Hst3p and Hst4p preserve genome integrity by controlling histone H3 lysine 56 deacetylation.. Curr Biol.

[70] Maas NL, Miller KM, DeFazio LG, Toczyski DP (2006). Cell cycle and checkpoint regulation of histone H3 K56 acetylation by Hst3 and Hst4.. Mol Cell.

[71] Thaminy S, Newcomb B, Kim J, Gatbonton T, Foss E (2007). Hst3 is regulated by Mec1-dependent proteolysis and controls the S phase checkpoint and sister chromatid cohesion by deacetylating histone H3 at lysine 56.. J Biol Chem.

[72] Lydeard JR, Jain S, Yamaguchi M, Haber JE (2007). Break-induced replication and telomerase-independent telomere maintenance require Pol32.. Nature.

[73] Burgers PM, Gerik KJ (1998). Structure and processivity of two forms of *Saccharomyces cerevisiae* DNA polymerase delta.. J Biol Chem.

[74] Smith CE, Lam AF, Symington LS (2009). Aberrant double-strand break repair resulting in half crossovers in mutants defective for Rad51 or the DNA polymerase delta complex.. Mol Cell Biol.

[75] Formosa T, Nittis T (1999). Dna2 mutants reveal interactions with DNA polymerase α and Ctf4, a Pol α accessory factor, and show that full Dna2 helicase activity is not essential for growth.. Genetics.

[76] Sikorski RS, Hieter P (1989). A system of shuttle vectors and yeast host strains designed for efficient manipulation of DNA in *Saccharomyces cerevisiae*.. Genetics.

[77] Brachmann CB, Davies A, Cost GJ, Caputo E, Li J (1998). Designer deletion strains derived from *Saccharomyces cerevisiae* S288C: a useful set of strains and plasmids for PCR-mediated gene disruption and other applications.. Yeast.

[78] Tong AHY, Boone C (2007). High-throughput strain construction and systematic synthetic lethal screening in *Saccharomyces cerevisiae*.. Meth Microbiol.

[79] Goldstein AL, McCusker JH (1999). Three new dominant drug resistance cassettes for gene disruption in *Saccharomyces cerevisiae*.. Yeast.

[80] Wach A, Brachat A, Pohlmann R, Philippsen P (1994). New heterologous modules for classical or PCR-based gene disruptions in *Saccharomyces cerevisiae*.. Yeast.

[81] Rothstein R (1991). Targeting, disruption, replacement, and allele rescue: integrative DNA transformation in yeast.. Methods Enzymol.

[82] Vallen EA, Hiller MA, Scherson TY, Rose MD (1992). Separate domains of KAR1 mediate distinct functions in mitosis and nuclear fusion.. J Cell Biol.

[83] Dershowitz A, Newlon CS (1993). The effect on chromosome stability of deleting replication origins.. Mol Cell Biol.

[84] Lea D, Coulson C (1949). The distribution of numbers of mutants in bacterial population.. J Genetics.

[85] Brewer BJ, Lockshon D, Fangman WL (1992). The arrest of replication forks in the rDNA of yeast occurs independently of transcription.. Cell.

[86] Theis JF, Newlon CS (2001). Two compound replication origins in *Saccharomyces cerevisiae* contain redundant origin recognition complex binding sites.. Mol Cell Biol.

[87] Newlon CS, Lipchitz LR, Collins I, Deshpande A, Devenish RJ (1991). Analysis of a circular derivative of *Saccharomyces cerevisiae* chromosome III: a physical map and identification and location of ARS elements.. Genetics.

[88] Zou L, Elledge SJ (2003). Sensing DNA damage through ATRIP recognition of RPA-ssDNA complexes.. Science.

[89] Kondo T, Wakayama T, Naiki T, Matsumoto K, Sugimoto K (2001). Recruitment of Mec1 and Ddc1 checkpoint proteins to double-strand breaks through distinct mechanisms.. Science.

[90] Majka J, Binz SK, Wold MS, Burgers PM (2006). Replication protein A directs loading of the DNA damage checkpoint clamp to 5′-DNA junctions.. J Biol Chem.

[91] Majka J, Burgers PM (2003). Yeast Rad17/Mec3/Ddc1: a sliding clamp for the DNA damage checkpoint.. Proc Natl Acad Sci U S A.

[92] Melo JA, Cohen J, Toczyski DP (2001). Two checkpoint complexes are independently recruited to sites of DNA damage in vivo.. Genes Dev.

[93] Wang H, Elledge SJ (2002). Genetic and physical interactions between DPB11 and DDC1 in the yeast DNA damage response pathway.. Genetics.

[94] Gangloff S, McDonald JP, Bendixen C, Arthur L, Rothstein R (1994). The yeast type I topoisomerase Top3 interacts with Sgs1, a DNA helicase homolog: a potential eukaryotic reverse gyrase.. Mol Cell Biol.

