# Supplementary figures and images for: The DNA Damage Response Pathway Contributes to the Stability of Chromosome III Derivatives Lacking Efficient Replicators
Source: PLoS Genet. 2010 Dec 2;6(12):e1001227. doi: 10.1371/journal.pgen.1001227 (PMC2996327; doi:10.1371/journal.pgen.1001227)

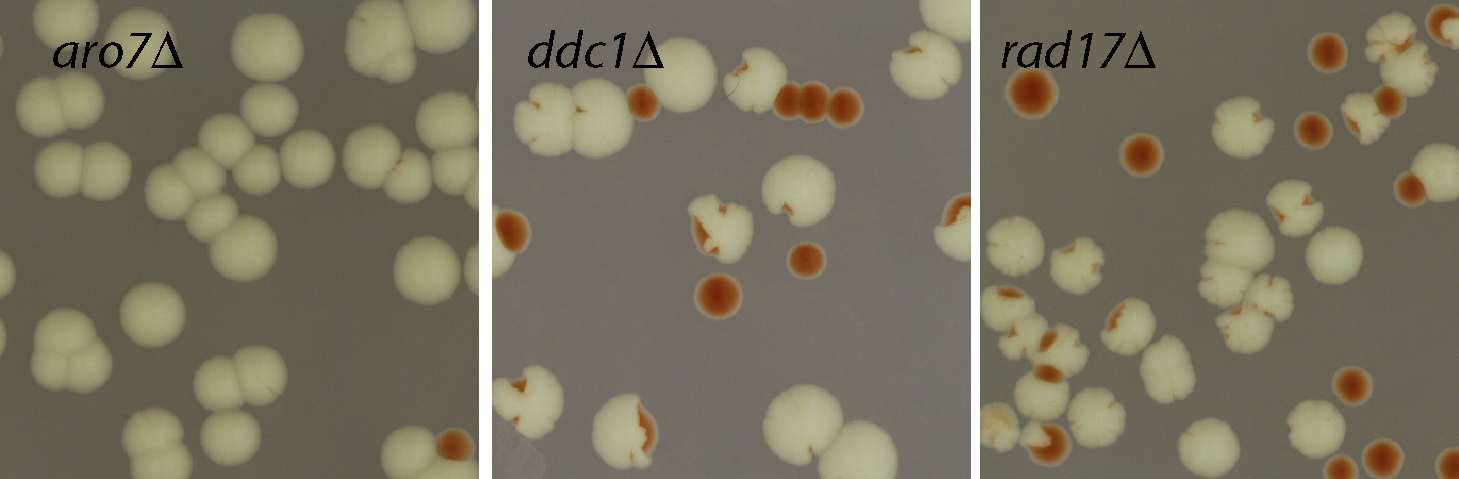

Supplement: Figure S1 — Sectoring patterns of aro7Δ, ddc1Δ, and rad17Δ strains. The 5ORIΔ-ΔR fragment was introduced into aro7Δ, ddc1Δ and rad17Δ strains by chromoduction, and the chromoductants were streaked on plates with limiting adenine and photographed after growth for 5 days. (1.70 MB TIF) [file pgen.1001227.s001.tif]

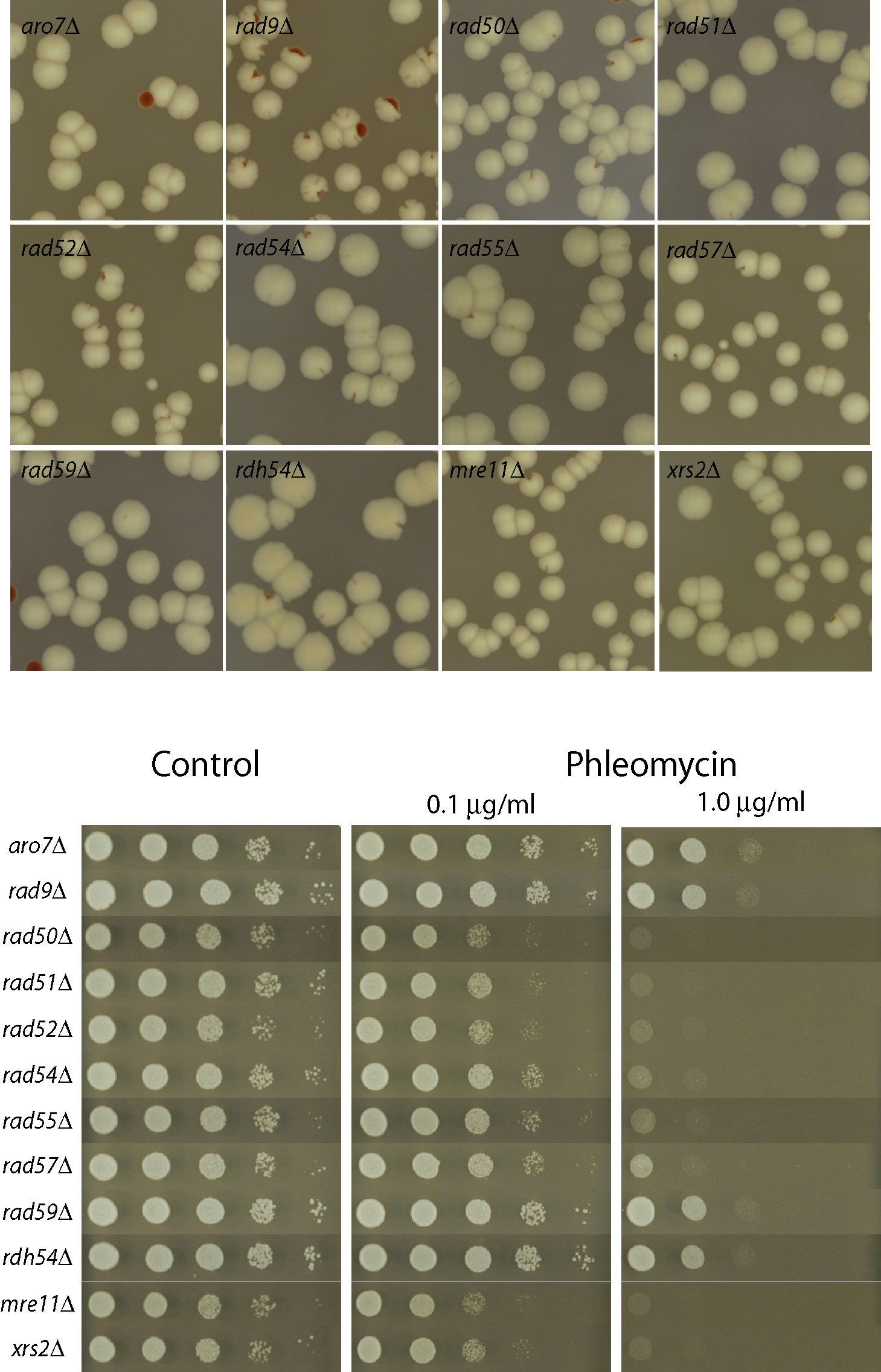

Supplement: Figure S2 — Sectoring patterns of mutants in the rad52 epistasis group. Top panels: 5ORIΔ-ΔR chromoductants of rad50Δ, rad51Δ, rad52Δ, rad54Δ, rad55Δ, rad57Δ, rad59Δ, rdh54Δ, mre11Δ and xrs2Δ strains isolated in the whole genome screen were streaked on plates with limiting adenine and photographed after growth for 5 days. aro7Δ and rad9Δ chromoductants were included as controls. Lower panels: Chromoductants were tested for sensitivity to phleomycin, which induces double-stranded breaks, to confirm that the strains carried the expected deletions. Cultures of the strains shown in the top panels were grown overnight in YEPD, serially diluted and spotted on YEPD plates (control) and plates with 0.1 and 1.0 µg/ml phleomycin. YEPD plates and 0.1 µg/ml phleomycin plates photographed after 3 days, 1.0 g/ml phleomycin plates after 5 days. Each of the strains, with the exception of rad59Δ and rdh54Δ, showed sensitivity, indicating that the sensitive strains carried the expected deletions. rad59 mutants have been reported to be 10,000-fold less sensitive to gamma irradiation than rad52 mutants [Bai et al], so the lack of sensitivity of the strain we tested was expected. The sensitivity of rdh54Δ to gamma irradiation had not been previously tested but it had been shown not to be sensitive to HO-induced double strand breaks [Klein et al]; we found that an authentic rdh54Δ mutant was also not sensitive to phleomycin. [Bai Y, Symington LS (1996) A Rad52 homolog is required for RAD51-independent mitotic recombination in Saccharomyces cerevisiae. Genes Dev 10: 2025–2037.] [Klein HL (1997) RDH54, a RAD54 homolog in Saccharomyces cerevisiae, is required for mitotic diploid-specific recombination and repair and for meiosis. Genetics 147: 1533–1543.] (6.45 MB TIF) [file pgen.1001227.s002.tif]
