# Supplementary material for: The DNA Damage Response Pathway Contributes to the Stability of Chromosome III Derivatives Lacking Efficient Replicators
Source: PLoS Genet. 2010 Dec 2;6(12):e1001227. doi: 10.1371/journal.pgen.1001227 (PMC2996327; doi:10.1371/journal.pgen.1001227)
Supplement: Table S1 — Results of secondary screen for Ofm mutants. (0.73 MB PDF) [file pgen.1001227.s003.pdf]

Table S1 Results of Secondary Screen for Ofm Mutants

High Confidence Ofm mutants

Strain	5ORIΔ-ΔR	5ORIΔ-ΔR	5ORIΔ-ΔR	5ORIΔ-ΔR	Sectors/colony 5ORIΔ-ΔR	Sectors/colony 0ORIΔ-ΔR	Comments
wild type control for primary screen *(aro7)*	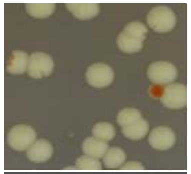	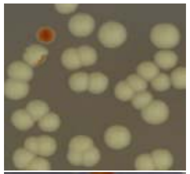	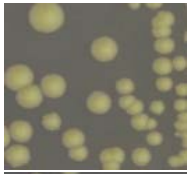	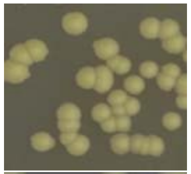	0-3	none	
asi2Δ	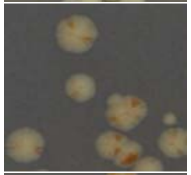	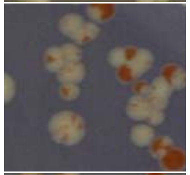	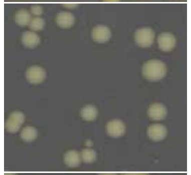	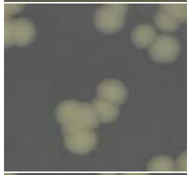	5-10	none	
ast2Δ	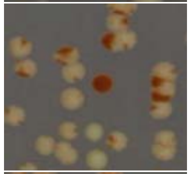	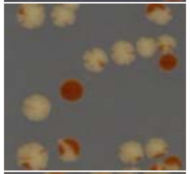	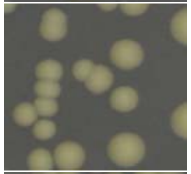	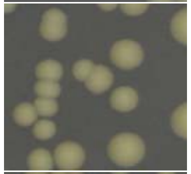	5-10	none	
bfa1Δ	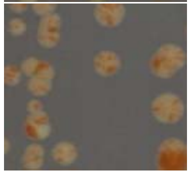	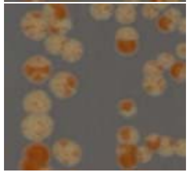	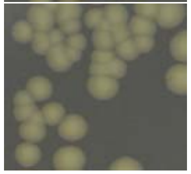	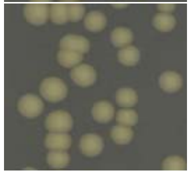	5-10	none	Also found in preliminary screen
bre5Δ	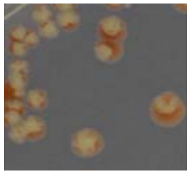	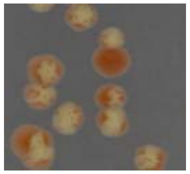	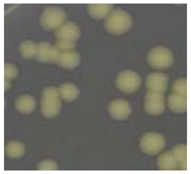	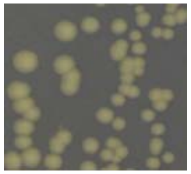	>10	rare	
chd1Δ	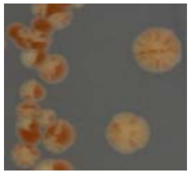	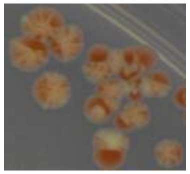	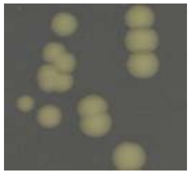	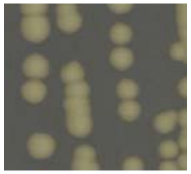	>10	none	Deletion moved into YKN10 background; Ofm phenotype confirmed
ctf18Δ	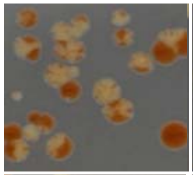	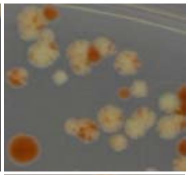	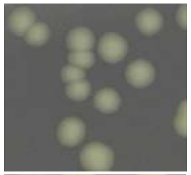	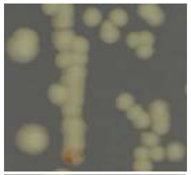	5-10	most colonies have none; rare highly sectored	Also found in preliminary screen

ecm7Δ	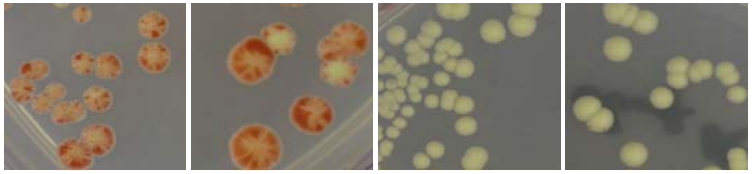	5->10	rare	
gut2Δ	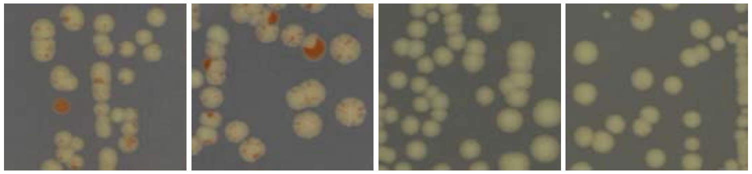	5-10	rare	
gyp1Δ	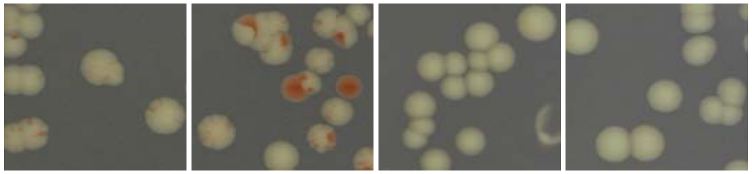	5-10 + some with no sectors	none	
hch1Δ	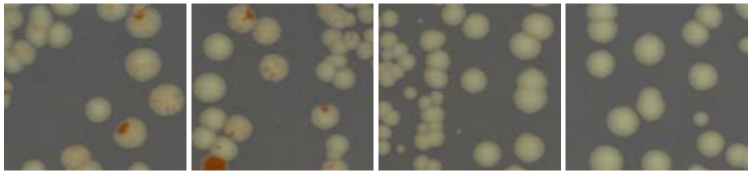	5-10	rare	
hst3Δ	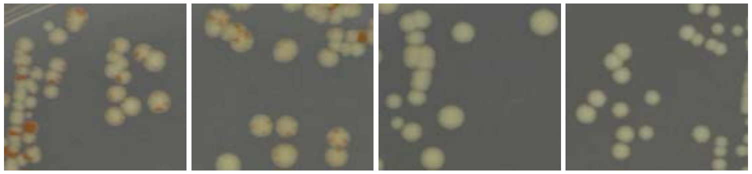	>10	none	Also found in preliminary screen
idh1Δ	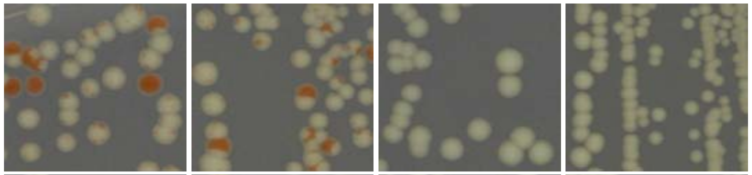	3-10	rare	
idh2Δ	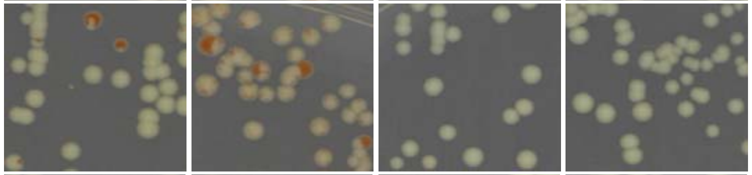	5-10	none	5ORIΔ chromoductants differ; *ircΔ* 5ORIΔ chromoductants show strong sectoring

ioc2Δ	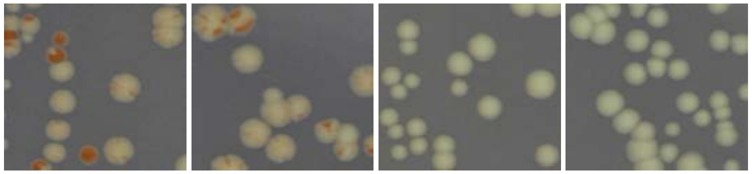	5-10	none	
irc14Δ	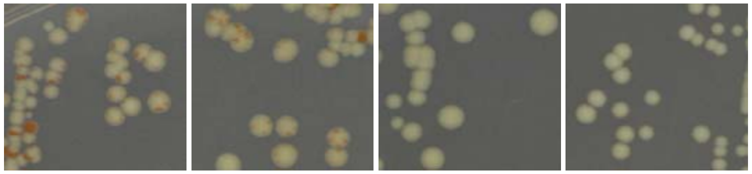			Dubious ORF that partially overlaps *IDH2*
isw1Δ	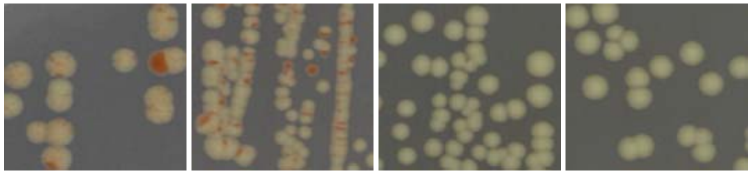	5-10; >10	rare; none	Deletion moved into YKN10 background; Ofm phenotype confirmed
lsm1Δ	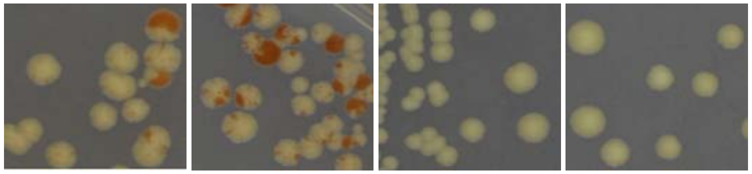	5->10	rare	
mad2Δ	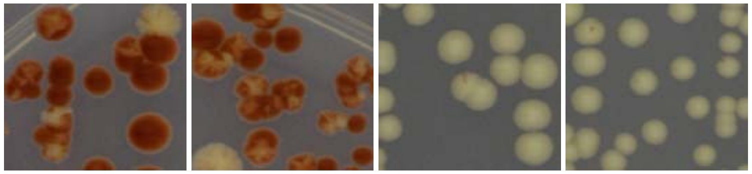	>10	a few colonies with a single sector	Also found in preliminary screen
mad3Δ	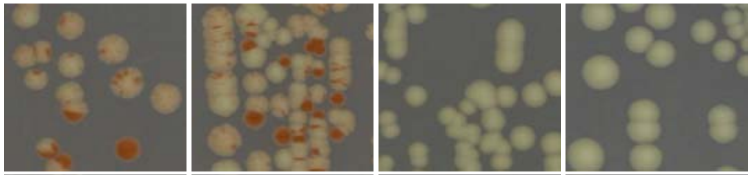	5->10	rare;none	
mcr1Δ	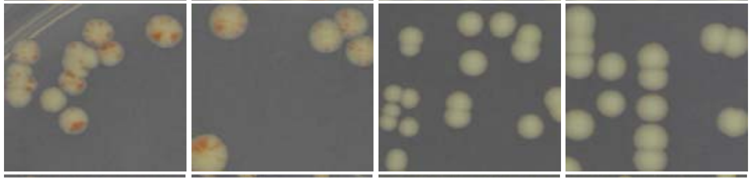	5-10	none	

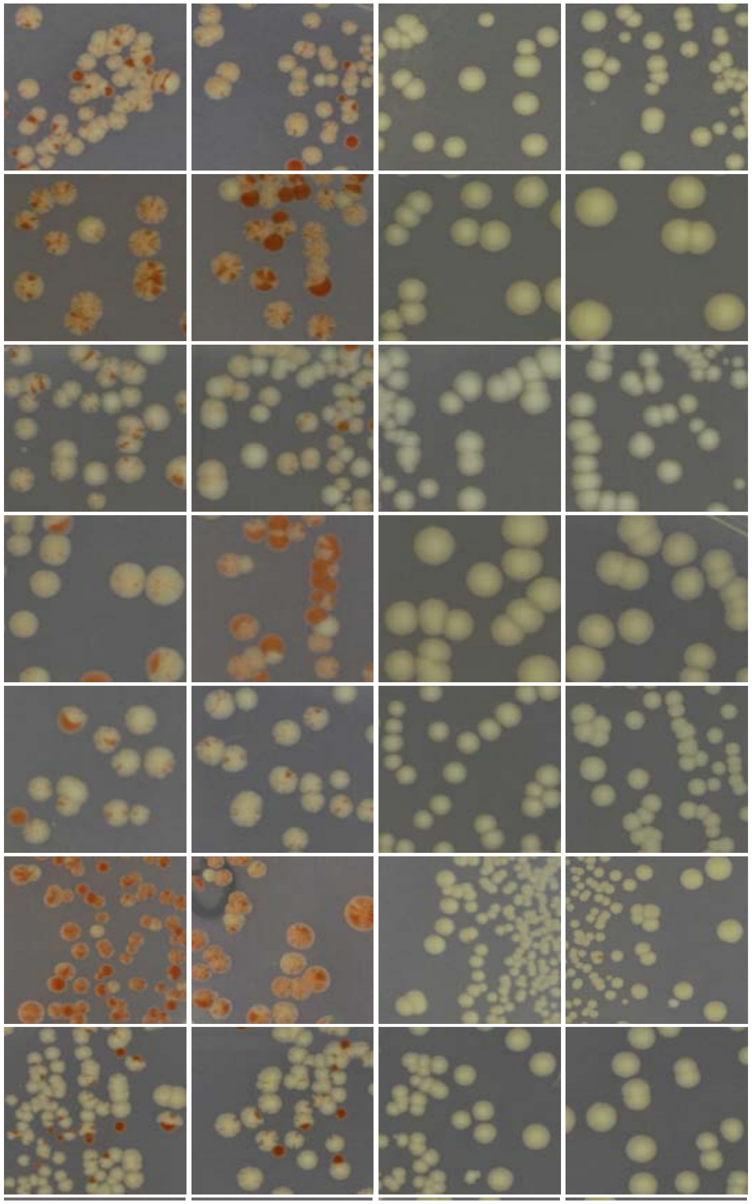

mid1Δ	5-10	none	
mtc1Δ	>10	rare	
mth1Δ	5-10 with some non-sectoring white colonies	none	
pol32Δ	>10; 5-10	rare	Deletion moved into YKN10 background; Ofm phenotype confirmed
puf3Δ	5-10	none	
rad9Δ			Pictures from preliminary screen. Deletion moved into YKN10 background; Ofm phenotype confirmed
rad9Δ	5-10	none	Deletion moved into YKN10 background; Ofm phenotype confirmed

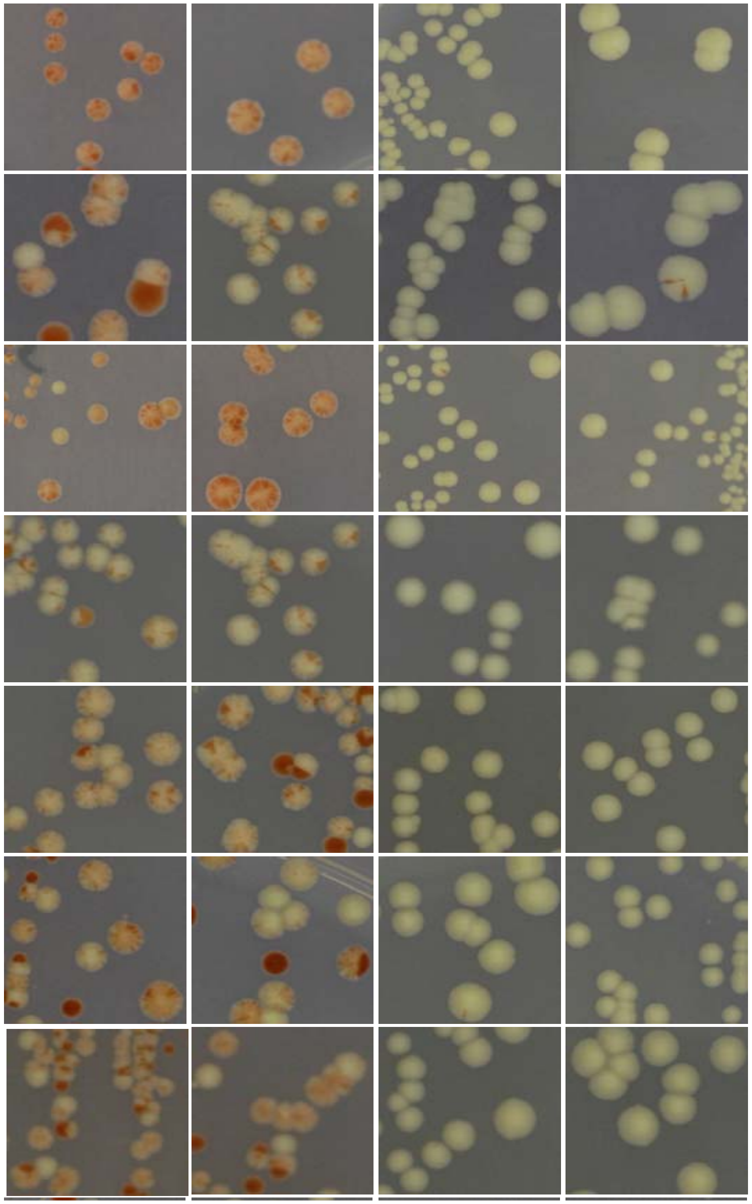

rad17Δ			Pictures from preliminary screen. Deletion moved into YKN10 background; Ofm phenotype confirmed
rad17Δ	5-10; >10	rare	Deletion moved into YKN10 background; Ofm phenotype confirmed
rad24Δ			Identified in preliminary screen. Deletion moved into YKN10 background; Ofm phenotype confirmed
rpa34Δ	5-10	none	
rpl20bΔ	>10	rare	
rpl34bΔ	5-10	rare	
sdp1Δ	5-10	rare	

sgf73Δ	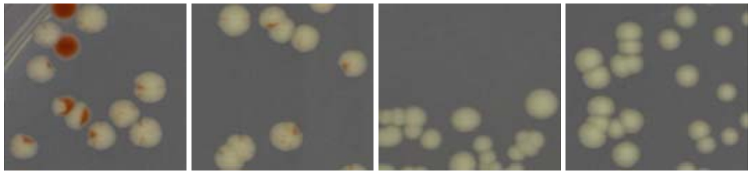	~5	none	
sgs1Δ	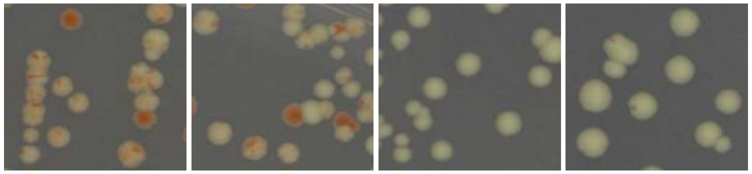	5-10	rare	Also found in preliminary screen.
sip3Δ	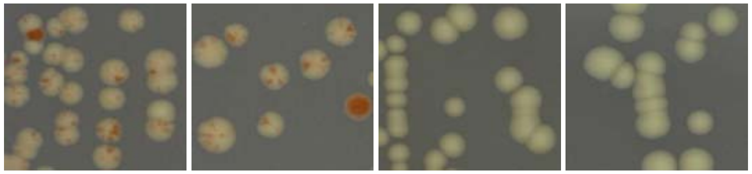	5-10	none	
skt5Δ	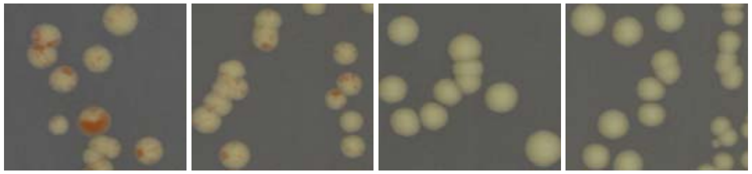	5-10	none	
sop4Δ	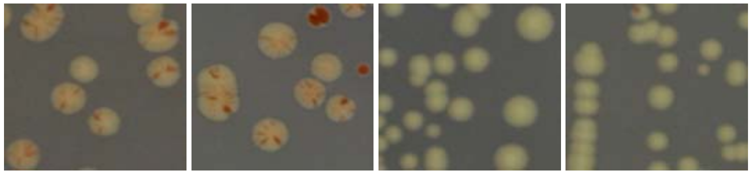	5-10	rare	
spe1Δ	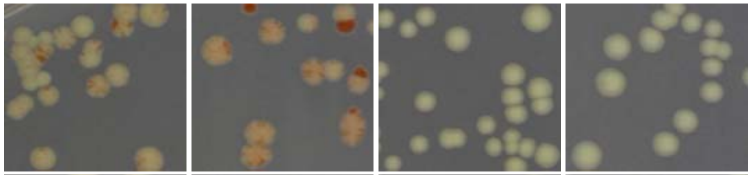	1-5; 5-10	none	
spt8Δ	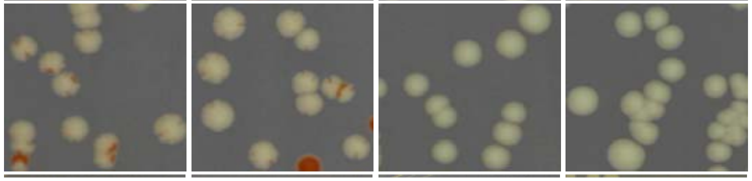	~5	none	

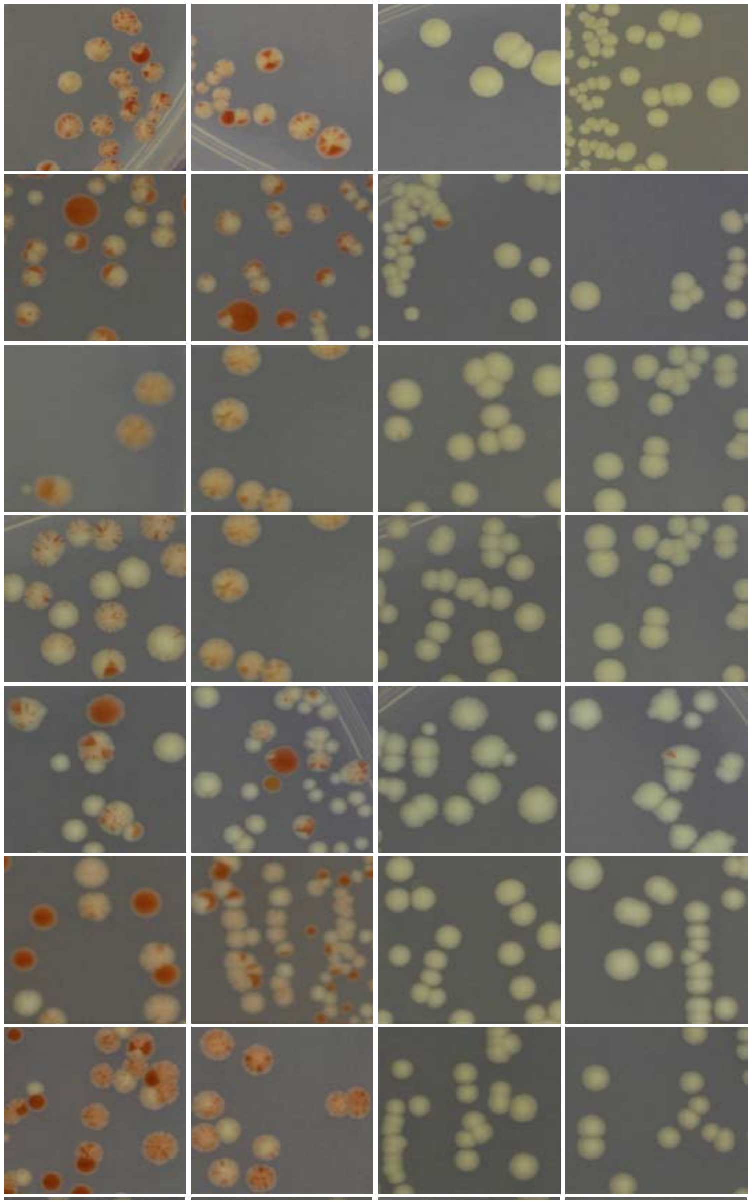

swi5Δ	5-10	none
ubp3Δ	>10	a few colonies with a single sector
vph2Δ	>10 plus some non-sectoring white colonies	rare
whi4Δ	>10	rare
ybr099cΔ	>10 plus petites	rare
ydr278cΔ	5-10	none
yer046w-aΔ	>10	rare

yfr016cΔ	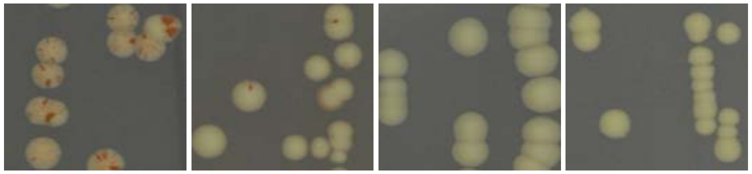	5-10 plus white non-sectoring colonies	none	
*yor024wΔ**	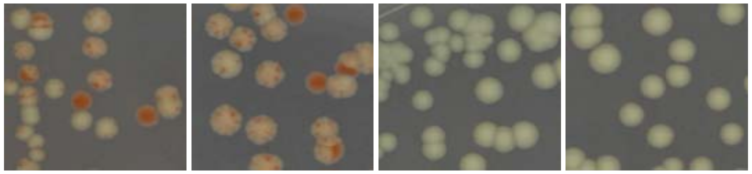	5-10 plus white non-sectoring colonies	none	YOR0124W is a dubious ORF upstream of *HST3*. The deletion leaves only 52-bp upstream of the *HST3* ORF and probably reduces *HST3* expression. *hst3Δ* chromoductants have a very similar phenotype.
ypk1Δ	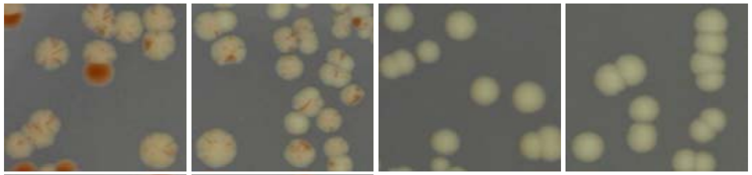	5-10 plus white non-sectoring colonies	none	
yhl005c2Δ	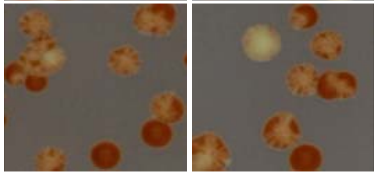	>10	none	*yhl005C* is a dubious ORF which partially overlaps *MRP4*, which encodes a mitochondrial small subunit ribosomal protein, and is upstream of *SHU1*, which functions in a *RAD51* and *RAD54*-dependent pathway for homologous recombination repair

Possible/Probable Ofm Mutants

acm1Δ	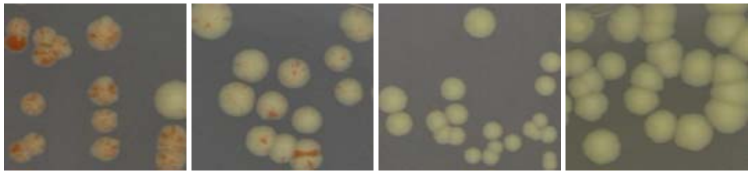	>10; 1-5	none	
bim1Δ	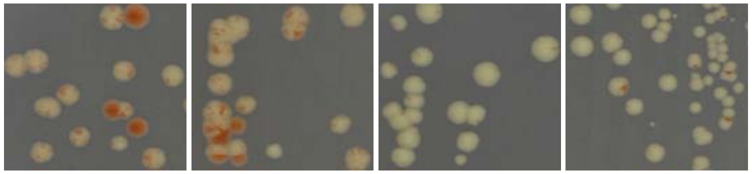	5-10	0-4	0ORIΔ chromoductants have different colony sectoring phenotypes

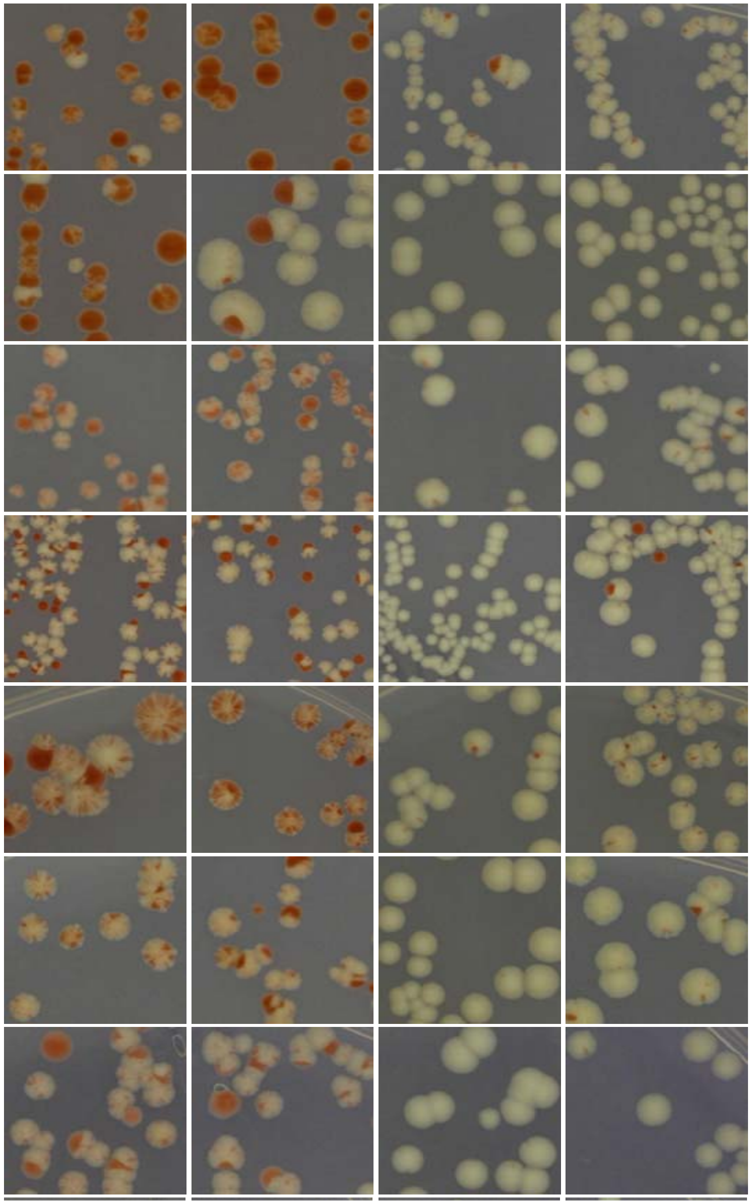

bub1Δ	>10	0-5	
bub2Δ	>10;	none	5ORIΔ chromoductants have different colony sectoring phenotypes
bub3Δ	>10	0-5	
chl1Δ	5-10	none; single sector in ~half of colonies	Also found in preliminary screen
csm3Δ	>10	1-10; rare	0ORIΔ chromoductants have different colony sectoring phenotypes
dia2Δ	>10	none; 1-5	0ORIΔ chromoductants have different colony sectoring phenotypes
rmi1Δ	5-10	small number of colonies with single sector	

rtg1Δ	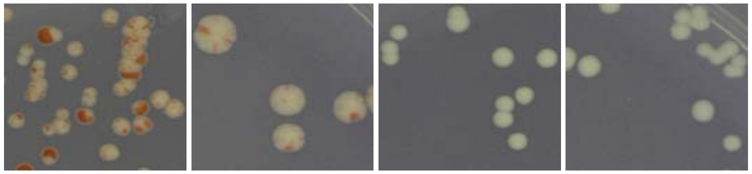	5-10	rare	Colonies are small, making sectoring phenotype difficult to score. One 5ORIΔ chromoductant has a mixture of sectoring and non-sectoring colonies.
rtg3Δ	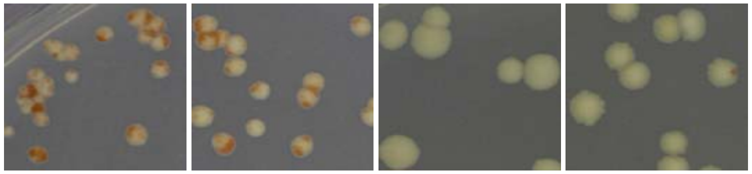	5-10	rare	Colonies of 5ORIΔ chromoductants are small, making sectoring phenotype difficult to score
sok2Δ	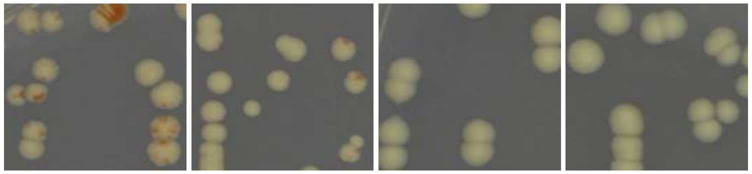	5-10; 1-5	none	5ORIΔ chromoductants have different colony sectoring phenotypes
tof1Δ	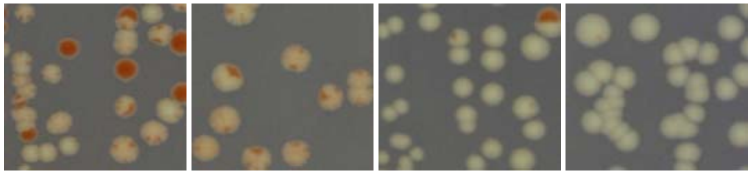	>10; 5-10	most colonies have single sector; rare	0ORIΔ chromoductants have different colony sectoring phenotypes
top3Δ	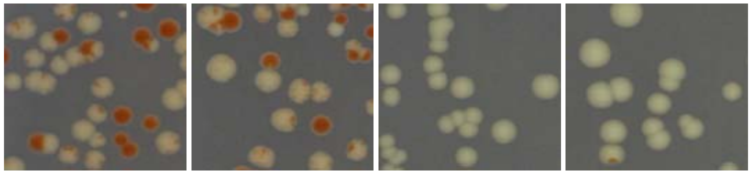	5-10	rare	Strain probably carries *sgs1* mutation, which suppresses slow growth phenotype of *top3*Δ. Phenotype similar to *sgs1*Δ strain, therefore is not informative about *top3*Δ phenotype.

Non-Ofm Mutants

ctf4Δ	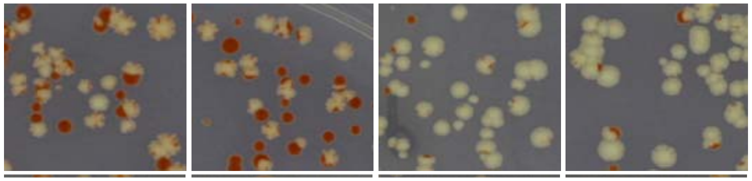	5-10	0->5

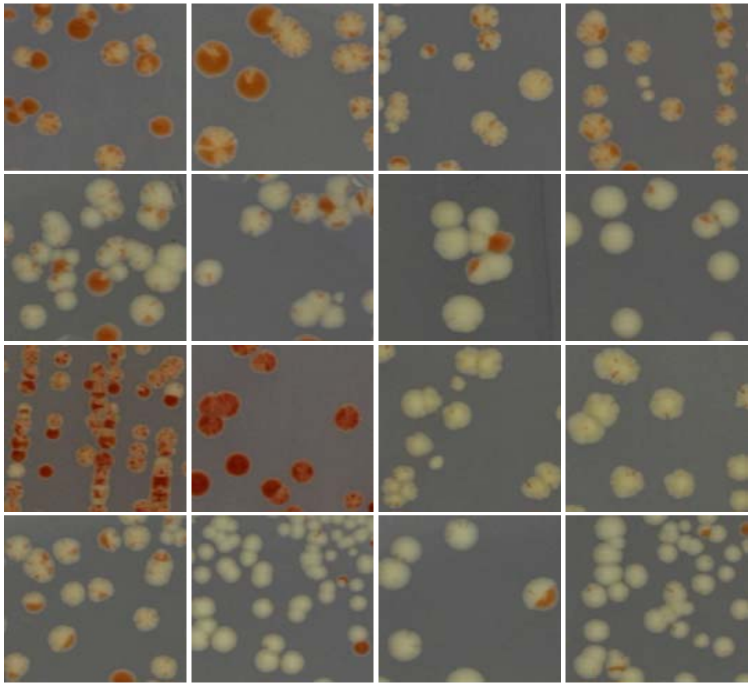

ctf8Δ	>10	>10
kar3Δ	1-10	0-5
mad1Δ	>10	0-5
sic1Δ	5-10	0-3
