# Supplementary material for: The DNA Damage Response Pathway Contributes to the Stability of Chromosome III Derivatives Lacking Efficient Replicators
Source: PLoS Genet. 2010 Dec 2;6(12):e1001227. doi: 10.1371/journal.pgen.1001227 (PMC2996327; doi:10.1371/journal.pgen.1001227)
Supplement: Table S2 — Strains used in this study. (0.06 MB DOC) [file pgen.1001227.s004.doc]

Table S2 Strains used in this study

Strains Genotype Reference

YKN10 *MAT***a** *HIS4 leu2-Δ1 ___* [1]

*ars305Δ his4-280 ars306Δ ars307Δ LEU2 C2G::SUP11-1*

*ars309Δ ars310Δ H9G::TRP1::Tel* **(5ORIΔ-ΔR)**

*ura3-52 lys2-801 trp1-Δ63 his3-Δ200 ade2-101 cyh2 can1*

*kar1-Δ15 ARO7*

Y7029 *MATα can1Δ::STE2pr-HIS3 lyp1Δ his3-Δ1 leu2-Δ0 ura3-Δ0*  [2]

YDN324 *mrc1Δ::NAT ars305Δ HIS4 leu2-Δ1 MAT***a**; *ura3-52; lys2-801;*

*trp1-Δ63; his3-Δ200; ade2-101; cyh2; can1; kar1-Δ15; ARO7*

YDN337 *ars305Δ HIS4 leu2-Δ1 MAT***a**;*ura3-52; lys2-801; trp1-Δ63; his3-Δ200;*

*ade2-101; cyh2; can1; kar1-Δ15; ARO7; bar1Δ::KAN*

CF4-16B *MAT***a** *his4-280; ade2-101; ura3-52* [3]

*MATα his4-290*

F510-3 *his4-280 MATα*____________________________________

*ars305Δ his4-280 ars306Δ ars307Δ LEU2 C2G::SUP11-1*

*ars309Δ ars310Δ H9G::TRP1::Tel* **(5ORIΔ-ΔR)**

*ade2-101; ura3-52; trp1-Δ63; rad52::URA3*

**Chromoduction recipient strains**

YJT242 *MATα can1Δ::STE2pr-HIS3 lyp1Δ his3-Δ1 leu2-Δ0 ura3-Δ0*

*ade2Δ::NAT* (Y7029 derivative)

YKN10R *MAT***a** *HIS4 leu2-Δ1; ura3-52; lys2-801; trp1-Δ63; his3-Δ200;*

*ade2-101; cyh2; can1; kar1-Δ15; ARO7*

YJT164 YKN10R *chk1Δ::KAN*

YIC110 YKN10R *mec1Δ::NAT sml1Δ::KAN*

YJT294 YKN10R *mrc1Δ::NAT*

YJT551 YKN10R *mrc1Δ::KAN*

YJT87 YKN10R *rad9* (*ofm14*)

YJT135 YKN10R *rad9Δ::KAN*

YJT167 YKN10R *rad17Δ::KAN*

YJT225 YKN10R *rad24Δ::KAN*

YIC111 YKN10R *rad53Δ::HIS3 sml1Δ::KAN*

YJT168 YKN10R *sml1Δ::KAN*

YJT540 *MAT***a** *HIS4 leu2-Δ1* *ura3-52 lys2-801 trp1-Δ63 his3-Δ367 ade2-101 cyh2 can1 kar1-Δ15 ARO7* *bar1-Δ1327 tel1Δ::URA3*

YJT547 YKN10R *mec1Δ::NAT sml1Δ::KAN* *tel1Δ::URA3*

YJT555 YKN10R *rad53Δ::HIS3 sml1Δ::KAN* *chk1Δ::NAT*

**Chromoduction Donor Strains** (all derivatives of CF4-16B)

F510-3-16 *MATα his4-290 LEU2 ___*

*ars305Δ his4-280 ars306Δ ars307Δ LEU2 C2G::SUP11-1*

*ars309Δ ars310Δ H9G::TRP1::Tel* **(5ORIΔ-ΔR)**

*ura3-52 trp1-Δ63 ade2-101*

F510α4A1-4 *MATα his4-290 LEU2 __ __ __*

*ars305Δ his4-280 ars306Δ ars307Δ LEU2 C2G::ADE2*

*ars309Δ ars310Δ H9G::TRP1::Tel* **(5ORIΔ-ΔR)**

*ura3-52 trp1-Δ63 ade2-101* *kar1-Δ15*

F013-1-24 *MATα his4-290 LEU2 _____*

*his4-280 LEU2 C2G::SUP11-1 H9G::TRP1::Tel* **(0ORIΔ-ΔR)**

*ura3-52 trp1-Δ63 ade2-101*

F013αB2C-1C *MATα his4-290 LEU2 __*

*his4-280 LEU2 C2G::ADE2 H9G::TRP1::Tel* **(0ORIΔ-ΔR)**

*ura3-52 trp1-Δ63 ade2-101* *kar1-Δ15*

YIC129 *his4-290 LEU2 MATα*

*ars305Δ his4-280 ars306Δ LEU2 C2G::SUP11-1 ars307Δ*

*ars309Δ ars310Δ MATα 307097::TRP1* **(5ORIΔ)**

*ura3-52 trp1-Δ63 ade2-101*

YJT296 *MATα his4-290 LEU2*

*mrc1Δ::NAT ars305Δ his4-280 ars306Δ ars307Δ LEU2 C2G::ADE2*

*ars309Δ ars310Δ H9G::TRP1::Tel* **(5ORIΔ-ΔR)**

*ura3-52 trp1-Δ63 ade2-101*

YJT366 *MATα his4-290 LEU2 _*

*mrc1Δ::NAT his4-280 LEU2 C2G::SUP11-1 H9G::TRP1::Tel* **(0ORIΔ-ΔR)**

*ura3-52; trp1-Δ63; ade2-101* [ρ°]

YJT373 *his4-290 LEU2 MATα*

*mrc1Δ::NAT ars305Δ his4-280 ars306Δ LEU2 C2G::SUP11-1*

*ars307Δ ars309Δ ars310Δ MATα 307097::TRP1* **(5ORIΔ)**

*ura3-52 trp1-Δ63 ade2-101*

YJT542 *his4-290 LEU2 MATα*

*D10B::Tel::NAT ars305Δ his4-280 ars306Δ LEU2 C2G::ADE2 ars307Δ CEN3::CEN4 (ars308Δ) ars309Δ ars310Δ MATα* **(ΔL-6ORIΔ)**

*ura3-52; trp1-Δ63; ade2-101*

1. Theis JF, Dershowitz A, Irene C, Maciariello C, Tobin ML, et al. (2007) Identification of mutations that decrease the stability of a fragment of *Saccharomyces cerevisiae* chromosome III lacking efficient replicators. Genetics 177: 1445-1458.

2. Tong AHY, Boone C (2007) High-throughput strain construction and systematic synthetic lethal screening in *Saccharomyces cerevisiae*. Meth Microbiol 36: 369-383.

3. Dershowitz A, Snyder M, Sbia M, Skurnick JH, Ong LY, et al. (2007) Linear derivatives of *Saccharomyces cerevisiae* chromosome III can be maintained in the absence of autonomously replicating sequence elements. Mol Cell Biol 27: 4652-4663.
