# Supplementary material for: The DNA Damage Response Pathway Contributes to the Stability of Chromosome III Derivatives Lacking Efficient Replicators
Source: PLoS Genet. 2010 Dec 2;6(12):e1001227. doi: 10.1371/journal.pgen.1001227 (PMC2996327; doi:10.1371/journal.pgen.1001227)
Supplement: Table S3 — Quantitation of 2D gels. (0.03 MB DOC) [file pgen.1001227.s005.doc]

**Table S3 Quantitation of 2D gels**

| Mutant | Strain | ARS element | % replication bubbles^a^ | % replication bubbles^b^ |
| --- | --- | --- | --- | --- |
| *rad9Δ* | YJT135 | *ARS301* | 6.26 | 4.36 |
| *rad9Δ* | YJT136 | *ARS301* | 1.67 | 6.30 |
| *rad9Δ* | YJT135 | *ARS303/303/320* | 5.93 | 7.64 |
| *rad9Δ* | YJT136 | *ARS303/303/320* | 2.29 | 2.27 |
| *mec1Δ sml1Δ* | YIC109 | *ARS301* | 12.06 | 14.55 |
| *mec1Δ sml1Δ* | YIC110 | *ARS301* | 7.40 | 13.60 |
| *mec1Δ sml1Δ* | YIC109 | *ARS303/303/320* | 9.37 | 13.63 |
| *mec1Δ sml1Δ* | YIC110 | *ARS303/303/320* | 15.71 | 15.37 |

^a^Calculated as signal in bubble/(signal in bubble + 0.5 signal in Y). Shapes were drawn around bubble and Y signals and signal above background was quantitated.

^b^Calculated as signal in bubble/(signal in bubble + 0.5 signal in Y). Based on landmarks present in the patterns, vertical lines were drawn at equivalent positions on the gels, and the intensities along those lines were graphed, areas under the peaks were integrated.
