# Supplementary material for: The DNA Damage Response Pathway Contributes to the Stability of Chromosome III Derivatives Lacking Efficient Replicators
Source: PLoS Genet. 2010 Dec 2;6(12):e1001227. doi: 10.1371/journal.pgen.1001227 (PMC2996327; doi:10.1371/journal.pgen.1001227)
Supplement: Table S4 — Appearance of rarely-sectored colonies in strains carrying the ΔL-6ORIΔ fragment. (0.03 MB DOC) [file pgen.1001227.s006.doc]

Table S4 Appearance of rarely-sectored colonies in strains carrying the ΔL-6ORIΔ fragment

| Strain | Rate x 10^5^ |
| --- | --- |
| Wild Type | ND^1,2^ |
| *mrc1Δ* | 640 ± 120 cloNAT^R^ |
| *rad24Δ* | 5100 ± 800 cloNAT^S^ |
| *rad9* | 3200 ± 600 cloNAT^S^ |
| *mec1Δ sml1Δ* | 530 ± 90 cloNAT^S^ |

^1^ ND, not determined

^2^ cloNAT^S^ Ade^+^ Leu^+^ cells, the most common rearrangement detected in the mutants, were readily identified by replica-plating, but technical limitations (distinguishing cloNAT^S^ Ade^+^ Leu^+^ sectors from cloNAT^S^ Ade^+^ Leu^+^ colonies by replica-plating) precluded successful measurement of a rate of appearance of cloNAT^S^ colonies by fluctuation analysis.
